# Network pharmacology refined with non-ubiquity and decoy-controlled molecular docking reveals insights into *Moringa oleifera* phytochemicals targeting insulin resistance

**DOI:** 10.3389/fbinf.2026.1756081

**Published:** 2026-03-10

**Authors:** Armi Katrina Santos-Enriquez, Fabian M. Dayrit, Armando Jerome de Jesus, Nina Rosario L. Rojas

**Affiliations:** 1 Department of Physical Sciences and Mathematics, College of Arts and Sciences, University of the Philippines Manila, Manila, Philippines; 2 Department of Chemistry, School of Science and Engineering, Ateneo De Manila University, Quezon City, Philippines

**Keywords:** *Moringa oleifera*, insulin resistance, network pharmacology, network visualization, molecular docking, non-ubiquity, decoys

## Abstract

*Moringa oleifera* phytochemicals were predicted to target insulin resistance proteins using a modified network pharmacology and molecular docking approach. Two hundred ninety *M. oleifera* phytochemicals with their aglycones, acetylase and myrosinase degradation products were compiled from literature and phytochemical databases. Nine protein targets were identified from the intersection of gene lists with high relevance to insulin resistance from GeneCards and DisGeNET and the target genes predicted by reverse screening using Swiss Target Prediction: protein-tyrosine phosphatase 1B (PTPN1), 11-beta-hydroxysteroid dehydrogenase 1 (HSD11B1), peroxisome proliferator-activated receptor α (PPARα), peroxisome proliferator-activated receptor γ (PPARγ), PI3-kinase p85-alpha subunit (PIK3R1), insulin receptor (INSR), tumor necrosis factor α (TNF-α), endothelial nitric oxide synthase (eNOS) and hepatic lipase (LIPC). Binding affinities of phytochemicals with the targets were predicted using Autodock Vina. The predicted binding affinities were classified according to calculated thresholds using receiver operating characteristic (ROC) calculations of binding affinities of: (a) binders (annotated drugs and other molecules with known interaction with each target), and (b) decoys (molecules not expected to bind to a specific target). In addition, ubiquitous phytochemicals were filtered out to differentiate the effect on insulin resistance of *M. oleifera* from that of other plants. The resulting phytochemical-protein interaction network was visualized using Cytoscape. All mentioned targets, except hepatic lipase, were key targets based on various network centrality measures. Previous studies on murine models have shown that isothiocyanate-rich *M. oleifera* extracts ameliorate insulin resistance. Using our approach, the following phytochemicals, with predicted moderate bioavailability, high GI absorption, and probable binding with insulin resistance targets, are recommended for further *in vivo* or *in vitro* validation for insulin resistance activity: boldione (a steroid); aurantiamide acetate and aurantiamide (peptide derivatives); O-ethyl-[(3,4-dihydroxyphenyl)methyl] carbamothioate and O-methyl-N-[(4-hydroxyphenyl)methyl] carbamothioate (thiocarbamates); 4α,6α-dihydroxyeudesman-8β,12-olide (a sesquiterpenoid); sanleng acid and tianshic acid (fatty acid derivatives); 2′,5,5′,7-tetrahydroxyflavone; 2′,3,5,7-tetrahydroxyflavone; and 6-hydroxykaempferol (flavonoids). By combining network centrality measures of targets, using ROC-derived thresholds for docking energies, and considering ubiquity of phytochemicals, our refined network pharmacology approach may aid in discovering key bioactive phytochemicals as potential chemical markers for standardization and differentiation of an herbal drug.

## Graphical Abstract

**Figure:**
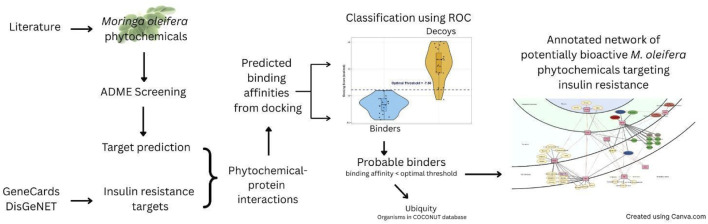

## 1 Introduction

The normal metabolic effect of insulin signaling results in glucose uptake and utilization in insulin-sensitive cells such as that of skeletal muscle, heart muscle, fat tissue, and the liver. Insulin resistance is defined as the inability or reduced ability of a known quantity of insulin to induce glucose uptake and utilization as compared to the normal population. It is part of a cluster of cardiovascular-metabolic abnormalities referred to as “metabolic syndrome” (Lebovitz, 2001), occurs earlier than cardiovascular disease or type II diabetes (Bodhini and Mohan, 2018), and has been found to be the main pathologic mechanism that puts individuals at risk for these diseases (Reddy et al., 2010). This is a major global health issue as prevalence of metabolic syndrome can be estimated to be about one-quarter of the world population (Saklayen, 2018; Fahed et al., 2020). This underscores the need to develop therapies for insulin resistance to prevent the progression of chronic diseases.

Current drugs used to treat insulin resistance include metformin, a biguanide, and thiazolidinediones (TZDs). Metformin has a well-established safety profile and relatively low cost, but also effects gastrointestinal disturbances in 30% of patients (LaMoia and Shulman, 2021). In rare cases, lactic acidosis due to metformin toxicity can occur, leading to central nervous system dysfunction, cardiovascular failure, renal failure, and death (Feng et al., 2022). Thiazolidinediones, on the other hand, have fallen out of favor because of concerns about heart failure and bone fractures (Lebovitz, 2019). Due to the side effects brought about by these drugs, there is a need for alternative or adjunct therapies to treat insulin resistance.

*Moringa oleifera* Lam. is known as the ben tree, drumstick tree, horseradish tree, and “malunggay” in the Philippines. Dubbed a “miracle tree,” all its parts have found uses in traditional medicine in various cultures for various ailments, while also being a valuable source of nutrition. Preclinical studies on the multiple pharmaceutical properties of *Moringa oleifera* include anti-inflammatory, antihypertensive, antidiabetic, antiatherosclerosis and hypolipidemic effects (Farooq et al., 2012). Various clinical studies have already demonstrated that *M. oleifera* has a favorable effect on glycemia markers, blood pressure or blood lipids compared to placebo, implying that it acts as a natural insulin sensitizer (Gómez-Martínez et al., 2022; Mthiyane et al., 2022). Recent studies have shown that *M. oleifera* positively modulates the protein expression of SIRT1 and PPARα *in vitro* (Duranti et al., 2021), and that it activates the AMPK signal pathway in db/db mice used as a model for type 2 diabetes mellitus (Bao et al., 2020). However, the active principles and the mechanisms by which *M. oleifera* phytochemicals act to reduce insulin resistance still need to be fully unraveled.

Numerous *M. oleifera* bioactive compounds have been recognized. Glucosinolates are relatively biologically inert, highly water-soluble compounds that are characteristic of cruciferous vegetables and related plant families including moringa (Fahey et al., 2019). Myrosinase (thioglucoside glucohydrolase) is released once the plant tissues are damaged, causing the rapid conversion of glucosinolates to isothiocyanates with concomitant removal of the glucose moiety. Other possible myrosinase degradation products include epithionitriles, thiocyanates, nitriles, isothiocyanates and oxazolidinethiones (Vaughn and Berhow, 2005).

Moringa isothiocyanates differ from other plant isothiocyanates by the presence of an aromatic ring and a rhamnose moiety (Dayal et al., 2013). It has been demonstrated that the administration of isothiocyanate-rich aqueous extract from *M. oleifera* leaves in mice fed with a very high fat diet reduced weight gain, insulin resistance and hepatic gluconeogenesis (Waterman et al., 2015). Hence it may be possible that active isothiocyanates may be responsible for the anti-insulin resistance activity.

One notable isothiocyanate is moringin, or 4-(α-L-rhamnosyloxy)benzyl isothiocyanate, formed from the bioactivation of glucomoringin with myrosinase (Galuppo et al., 2014). Moringin has been identified as an active antimicrobial agent against several bacteria and fungi (Eilert et al., 1981), shown to counteract the inflammatory cascade in neurodegenerative diseases (Galuppo et al., 2014; Jaafaru et al., 2018), and to have activity against human malignant astrocytoma (brain cancer) cells (Rajan et al., 2016).

Other active compounds include thiocarbamates such as niaziminin, one of the compounds responsible for the blood-pressure lowering effect of *M. oleifera* in rats, along with the 4′-O-acetylated derivative of moringin (Faizi et al., 1994). In a mouse study, it was demonstrated that carbohydrate and lipid metabolism was improved by niazirin, another thiocarbamate (Bao et al., 2020). Furthermore, it was determined that moringin, niazimicin, and β-sitosterol-3-O-β-D-glucopyranoside from seeds exhibit significant antitumor promoting activity, with niazimicin, a thiocarbamate glycoside, being the most potent among them (Guevara et al., 1999).

Other authors point out that flavonoids and phenolics, such as quercetin, rutin, chlorogenic acid, and neochlorogenic acid, have also been reported to exhibit antidiabetic activity. These have been proposed to exert antidiabetic effects by inhibition of digestive enzymes, exerting protective antioxidant mechanism by removal of reactive oxygen species, inhibition of carbohydrate digestive enzymes, stimulation of glucose uptake into cells, inhibition of AMPK signaling pathway, and inhibition of cytokine-induced signaling pathway (Habtemariam, 2017; Vergara-Jimenez et al., 2017). As there are numerous bioactive compounds present in *M. oleifera*, this body of evidence suggests that the Moringa phytochemicals may act through various mechanisms to increase insulin sensitivity.

Network pharmacology is a network-based view of drug action approach that attempts to understand drug interactions with multiple targets that form part of a biological network (Hopkins, 2007; Yildirim et al., 2007). The usefulness of network pharmacology has emerged because of the convergence of different techniques, such as network analytics tools, high resolution mass spectrometry, molecular modelling, metabolomics, and various bioinformatics and chemical databases. Network pharmacology has been touted as an important breakthrough in the study of medicinal plants and natural products, particularly in offering a systematic way to reveal the pharmacologic mechanisms of medicinal plants formulas (Zhang et al., 2013; Yuan et al., 2017; Noor et al., 2022; Wang et al., 2022; Wu et al., 2022; Zhao et al., 2023). Network pharmacology is particularly useful for complex diseases, allowing discovery of numerous relationships between natural products and multiple targets associated with a disease. This may reduce the need for iterative trial-and-error isolation and bioassay. Validation of the results gleaned from network pharmacology is still required, and research in this area has seen positive development with *in vitro* or *in vivo* validation increasingly being integrated to confirm the findings from *in silico* methods. However, there is also a pressing need for standardization of *in silico* methods to ensure accuracy, data quality, completeness, and consistency.

Other researchers have used network pharmacology in conjunction with cell-based assays to understand insulin resistance activity of *M. oleifera* seeds (Huang et al., 2020). Their study suggests that glycosidic isothiocyanates and glycosidic benzylamines acted on key targets, such as SRC, PTPN1, and CASP3. Similar studies have been used to understand its hypoglycemic effect and identified robinetin as a key active phytochemical (Hong et al., 2023).

Receiver operating characteristic (ROC) curve is a graphical plot that illustrates the performance of a binary classifier model. It has previously been used by various researchers for evaluation of docking protocols to screen inhibitors of an identified target (Pathania et al., 2013) and to compare the performance of different docking programs for specific targets (Deng and Verlinde, 2008; Shamsian et al., 2023). It was used to evaluate the performance of systemsDock, a web server for network pharmacology-based prediction (Hsin et al., 2016). ROC has also been used to identify hub genes from high-throughput gene expression datasets that are differentially expressed between patients with disease and healthy individuals (Wang et al., 2021; Gao et al., 2025; Hu et al., 2025).

This study aims to reveal the anti-insulin resistance phytochemicals present in *M. oleifera*, Lam. using a network pharmacology and docking approach which stratifies interactions based on enrichment of binding affinity of annotated binders *versus* nonbinders or decoys. This strategy increases confidence in the predictions made using network pharmacology as it does not simply accept negative docking scores as predictors for binding. Instead, based on ROC calculations, interactions between phytochemicals and targets are classified as probable or nonprobable if the affinity is lower or higher than the optimal threshold.

Moreover, although there are numerous phytochemicals that are considered ubiquitous across different plants, it is well known that plants exert different effects or activities. Although these ubiquitous phytochemicals may also influence the disease of interest, it would be helpful to distinguish non-ubiquitous *M. oleifera* phytochemicals that influence insulin resistance. This study also employs network visualization that incorporates subcellular location, phytochemical class, and pharmacokinetic properties that highlight specific interactions (Briones et al., 2021). Aside from rationalizing the anti-insulin resistance mechanism of *M. oleifera*, the results of this study may aid in establishing key bioactives that may be used as chemical markers for the standardization of an herbal drug.

## 2 Methods

### 2.1 Phytochemicals identification and screening

*Moringa oleifera* phytochemicals were compiled from literature (Anwar et al., 2007; Moyo et al., 2011; Nadeem and Imran, 2016; Wang et al., 2016; Abd Rani et al., 2018; Lin et al., 2019; Huang et al., 2020; Hong et al., 2023) and the Indian Medicinal Plants, Phytochemistry and Therapeutics (IMPPAT) database (https://cb.imsc.res.in/imppat/) (Mohanraj et al., 2018). For phytochemicals obtained from IMPPAT, only secondary metabolites found in the whole plant or all parts were considered. In addition, hydrolysis of glycosides was simulated by drawing the structures of their respective aglycones in ACD/ChemSketch 2021.2.1 (Advanced Chemistry Development, Inc. ACD/Labs, 2021). Other types of processing were also considered such as removal of acetyl groups, as well as thiocyanate, nitrile and isothiocyanate products from the enzymatic hydrolysis of glucosinolates by myrosinase (Vaughn and Berhow, 2005). Structural information in SMILES (Simplified Molecular Input Line Entry Specification) format was obtained from the PubChem database (https://pubchem.ncbi.nlm.nih.gov/) (Kim et al., 2023). For compounds that were not readily available in PubChem, structures were drawn manually, and SMILES were generated using ACD/ChemSketch. OpenBabel 3.1.1 (https://sourceforge.net/projects/openbabel/) (O’Boyle et al., 2011) was employed to check for duplicates.

SwissADME (http://www.swissadme.ch/) (Daina et al., 2017) was used to calculate the pharmacokinetic parameters of the phytochemicals. Phytochemicals that passed at least one of the drug-likeness criteria rules (Lipinski, Ghose, Veber, Egan or Muegge), and with no more than 1 PAINS alert (Pan-Assay Interference Compounds) were included. R 4.3.2 (https://www.R-project.org/) (R Core Team, 2021) with RStudio 2024.04.2 (https://posit.co/products/open-source/rstudio/) (RStudio, 2020) were used for semi-automation of tasks such as retrieving SMILES and Structured Data File (SDF) structures from PubChem, batch-wise submission of SMILES to Swiss Target Prediction and SwissADME. AI-based coding assistance was provided by Cody (https://sourcegraph.com/cody) (Sourcegraph, 2023).

### 2.2 Target identification

Prediction of protein targets of these phytochemicals was performed using Swiss Target Prediction (http://www.swisstargetprediction.ch/) (Daina et al., 2019). A study on nine target prediction web services assessed that Swiss Target Prediction produced the most reliable predictions while enriching more targets. Included among the tools investigated by Ji and co-workers are Swiss Target Prediction, Similarity Ensemble Approach (SEA), Polypharmacology Browser (PPB), PPB2 with four different target fishing methods, ChEMBL, and TargetNet (Ji et al., 2023). All targets with zero probability scores were removed.

Functional enrichment analysis of the gene lists of the predicted targets was performed using g:Profiler (https://biit.cs.ut.ee/gprofiler/gost) (Kolberg et al., 2023). Gene list relevant only to the disease of interest was collated using GeneCards (https://www.genecards.org/) (Stelzer et al., 2016) with the search term “insulin resistance” and a relevance score cutoff ≥12, then intersected with genes from DisGeNET (https://www.disgenet.com/) (Piñero et al., 2020), also using the search term “insulin resistance.” The Uniprot ID of the target was used as input in the Uniprot database (https://www.uniprot.org/) (The UniProt Consortium, 2023) to determine the subcellular location of each target. For targets with more than one location, the most accessible or external location was considered. For example, if the protein is in both cytoplasm and nucleus, then location would be tagged as “cytoplasm.” If it is both secreted and in the plasma membrane, then location would be tagged as “secreted.”

### 2.3 Molecular docking

The three-dimensional structures of ligands in SDF format were obtained from PubChem if available, otherwise, they were generated using ACD/ChemSketch. Preparation of the ligands was performed using OpenBabel, first by addition of hydrogens at pH 7.4, conversion to MOL2 format, energy minimization using the MMFF94 force field with a cutoff of 5,000 steps and then conversion to PDBQT format. The Uniprot IDs of the predicted targets were used to search the Protein Data Bank (PDB) (https://www.rcsb.org/) (Berman et al., 2000), and the PDB files with highest resolution, no mutations (wild type), and with a small molecule bound ligand was downloaded. The PDB files used for docking are listed in Table 1. The ligand’s cubic box length was set to 22.5 Å or 2.9 times the radius of gyration, whichever is higher, based on a prior study on the calculation of optimal box size for docking and virtual screening (Feinstein and Brylinski, 2015). The central coordinates of the box were set to that of the co-crystallized ligand in the PDB file. Preparation of the protein targets was performed by first removing bound ligands, waters, and ions then performing the Dock Prep command in Chimera 1.16 (https://www.cgl.ucsf.edu/chimera/) (Pettersen et al., 2004). Default parameters were used to complete side chains, and the file was saved in PDB format. Next, polar hydrogens and Gastgeiger charges were added and the file was saved as PDBQT using the prepare-receptor4. py script of MGL Tools 1.5.7 (https://ccsb.scripps.edu/mgltools/) (Morris et al., 2009). Docking was performed using Autodock Vina 1.1.2 (https://vina.scripps.edu/) (Eberhardt et al., 2021; Trott and Olson, 2010). The energy range, or the maximum energy difference between the best and worst binding modes, was increased to 4 kcal/mol from the default value of 3 kcal/mol to include other binding modes in the output (AutoDock Vina 1.12 Manual, 2011; Autodock Vina 1.2.0 documentation, 2021). Exhaustiveness is the number of initial random runs used for ligand conformation search and optimization (Che et al., 2023), and represents the amount of computational effort used during the docking experiment. A prior study showed that the default exhaustiveness value of 8 performs well for most box sizes, and that there is only minimal gain in accuracy with exhaustiveness greater than 25 (Agarwal and Smith, 2023). For this study, the exhaustiveness was increased from the default value of 8–16 to increase accuracy without compromising the capability of the hardware. Semi-automation of docking of all respective ligands with each receptor after preparation was accomplished using R with RStudio.

**TABLE 1:** Relevant insulin resistance targets.

Cluster	Target	Target name	Uniprot ID	PDB ID	Resolution (Å)	Location
Negative regulation of lipid storage	HSD11B1	11-beta-hydroxysteroid dehydrogenase 1	P28845	4YYZ	3.2	Endoplasmic reticulum membrane
	LIPC	Hepatic lipase	P11150	6OB0	2.81	Secreted
	NOS3	Endothelial nitric oxide synthase	P29474	3NOS	2.4	Cell membrane
	PPARA	Peroxisome proliferator-activated receptor alpha	Q07869	7E5I	1.58	Nucleus
	PPARG	Peroxisome proliferator-activated receptor gamma	P37231	7AWC	1.74	Cytoplasm
	TNF	TNF-alpha	P01375	7JRA	2.1	Secreted
Phosphatidyl-inositol 3-kinase complex, class I, and Insulin receptor complex	INSR	Insulin receptor	P06213	5HHW	1.79	Cell membrane
	PIK3R1	PI3-kinase p85-alpha subunit	P27986	5XGI	2.56	Cell membrane
	PTPN1	Protein-tyrosine phosphatase 1B	P18031	5K9W	2.01	Endoplasmic reticulum membrane

The docking score was taken as the average of two trials of the calculated binding affinity in kcal/mol. To validate the search space for structures of proteins with co-crystallized ligands, the ligands were redocked in the protein and the position of redocked ligands were visually compared to the position in the original crystal. Visualization of interactions between the ligands and targets was accomplished in 3D using ChimeraX (https://www.rbvi.ucsf.edu/chimerax) (Meng et al., 2023) and in 2D using LigPlot+ 2.2 (https://www.ebi.ac.uk/thornton-srv/software/LigPlus/) (Laskowski and Swindells, 2011).

### 2.4 Classification of predicted binding affinities

Binders used as positive controls were chosen from approved, investigational or experimental drugs for the targets from DrugBank (https://go.drugbank.com/) (Knox et al., 2024), including phytochemicals with probability prediction of 1 from Swiss Target Prediction (Briones et al., 2021), redocked ligands in the original crystal structure from the Protein Data Bank, and molecules from Binding DB (http://www.bindingdb.org/) (Gilson et al., 2016). Decoys or nonbinders used as negative controls were molecules that do not target insulin resistance proteins based on Swiss Target Prediction results, and decoys generated from the SMILES of positive controls using DUDE-Z (https://tldr.docking.org/start/dudez) (Stein et al., 2021). The optimal or best accuracy threshold score for each target was calculated in RStudio using the R package pROC 1.18.5 in R (Robin et al., 2011). Interactions were classified as either probable binders or probable nonbinders if the average docking score was below or above the optimal threshold score, respectively. Combining, filtering, retrieving, and plotting results were accomplished using R with RStudio.

### 2.5 Network construction and analysis

The Uniprot ID of the targets were inputted into the STRING database (https://string-db.org/) (Szklarczyk et al., 2023) with *Homo sapiens* as the organism. The interactions were downloaded as a TSV file. Phytochemical-protein target interactions and STRING interactions were merged in Cytoscape 3.10.3 (https://cytoscape.org/) (Shannon et al., 2003). Network centrality measures were calculated using the Analyze Network command of Cytoscape. Visualization integrated subcellular location, phytochemical class, docking score, and pharmacokinetic parameters (Briones et al., 2021).

Ubiquity of active phytochemicals was taken into consideration after the network was constructed to distinguish the effect of *M. oleifera* phytochemicals from that of other plants. To evaluate ubiquity of the phytochemicals, the SMILES of each was looked up in the Collection of Open Natural Products or COCONUT database (https://coconut.naturalproducts.net/) (Sorokina et al., 2021). If the phytochemical is unavailable on the COCONUT database, then the IMPPAT database was searched by name. The phytochemicals were arranged according to the number of occurrences in various organisms or species. Phytochemicals were considered as ubiquitous if the number of occurrences is greater than or equal to that of quinic acid, which is present in *M. oleifera* and a precursor in the shikimic acid pathway that occurs in all plants.

## 3 Results

A total of 290 *M. oleifera* phytochemicals, including aglycones (non-sugar unit), deacetylation products, and degradation products of glucosinolates by myrosinase were compiled and listed in Supplementary Table S1. It includes glucosinolates, isothiocyanates, nitriles, thiocarbamates, thiocyanates, benzenoid acids, esters, and phenols, alkaloids, terpenes, flavonoids, sterols, and fatty acid derivatives.

SwissADME was not able to provide results for two glycosylated flavonoids that were too large based on the character limit for SMILES, namely kaempferol-3-O-[ß-glucosyl-(1→2)]-[α-rhamnosyl-(1→6)]-ß-glucoside-7-O-α-rhamnoside and kaempferol-3-O-[α-rhamnosyl-(1→2)]-[α-rhamnosyl-(1→4)]ß-glucoside-7-O-α-rhamnoside. Null values in the Water Solubility, Pharmacokinetics, and Drug-likeness sections of SwissADME results were also obtained for linoleic acid, α-linolenic acid, 1,2,3,-triolein, sitosteryl linoleate, 1,2,3-trilinolein, 1,3-dilinoleoyl-2-olein, 1,3-dioleoyl-2-linolein, 1-linolenoylglycerol, 2-monolinolein, γ-linolenic acid, all-*E*-lutein, all-*E*-luteoxanthin, 13-*cis*-lutein, 15-*cis*-ß-carotene, all-*E*-zeaxanthin, α-carotene, violaxanthin, and all-*trans*-neoxanthin, and these compounds were excluded in the next steps.

ADME criteria are integral steps of the drug discovery process to increase the likelihood of clinical success and minimize wastage of resources that would have been spent on failures. Compounds that exhibit poor ADME parameters are typically excluded early in the evaluation process.

In the current study, phytochemicals that passed at least one of the drug-likeness criteria rules (Lipinski, Ghose, Veber, Egan or Muegge) based on calculations by SwissADME were included. Previous network pharmacology studies typically exclude certain phytochemicals in the succeeding steps based on certain criteria such as calculated drug-likeness score or oral bioavailability (Wang et al., 2022; Huang et al., 2023). In SwissADME, criteria for lipophilicity, size or molecular weight, polarity, water insolubility, unsaturation, and number of rotatable bonds (flexibility) are presented as an easily understandable radar chart. Sinalbin, a glucosinolate, was one of the identified *M. oleifera* phytochemicals. Sinalbin would have been excluded based on its calculated topological polar surface area (TPSA) of 220.02 Å^2^, which is beyond the cutoff of between 20 and 130 Å^2^ for drug-like substances in SwissADME (Daina et al., 2017). Having a high TPSA, it may be considered as too polar and was predicted to have low gastrointestinal absorption. However, a previous *in vivo* study using pigs showed the presence of sinalbin in the portal vein after feeding glucosinolates (Frandsen et al., 2011). This indicates that results of *in silico* ADME predictions in the initial stages of a network pharmacology study should not be used as strict cutoffs but rather as guidelines. Sinalbin did not pass the Lipinski, Veber, Egan or Muegge criteria for drug-likeness, but passed the Ghose criteria. Out of 290 initial phytochemicals, 168 were submitted to Swiss Target Prediction and the succeeding steps. Pharmacokinetic criteria will be more apparent in the network visualization that will be described later.

Of note, some recent network pharmacology studies have employed a metabolomic approach rather than ADME predictions to screen active phytochemicals that are absorbed. For example, when *Cyclocarya paliurus* leaf tea was administered to lipid-loaded mice, 13 phytoconstituents were determined to be present in the serum and included in the network pharmacology study (Zhai et al., 2018). Although there is a growing trend for the incorporation of metabolomics, even multi-omics, with network pharmacology, ADME calculations remain relevant as these are crucial for understanding how drugs behave in the body. Experimental ADME studies are a challenging and time-consuming aspect of the drug discovery pipeline and *in silico* calculations are usually undertaken as a first step.

**FIGURE 1:**
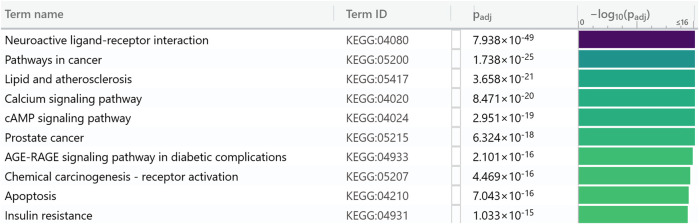
Top KEGG pathways targeted by *Moringa oleifera* phytochemicals from gProfiler.

Swiss Target Prediction is a ligand-based reverse screening method that predicts targets of molecules based on 2D and 3D shape similarity to ligands with known protein binding data in the ChEMBL database. Swiss Target Prediction predicted 8,164 phytochemical-protein interactions with nonzero probability for 918 targets of *M. oleifera* phytochemicals (Supplementary Table S2). Enrichment analysis using g:Profiler showed that insulin resistance is one of the top KEGG pathways targeted by *M. oleifera* phytochemicals (Figure 1).

**FIGURE 2:**
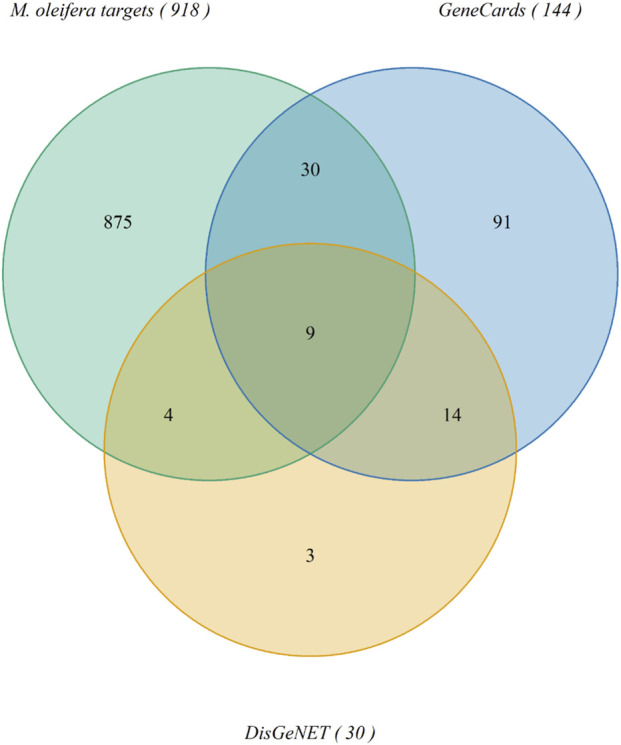
Venn diagram showing the number of targets of *Moringa oleifera* phytochemicals from Swiss Target Prediction intersected with the top insulin resistance genes from GeneCards and insulin resistance genes from DisGeNET. Image created using VennDiagram in RStudio.

The lists of insulin resistance genes were downloaded from GeneCards (Supplementary Table S3) and DisGeNET (Supplementary Table S4). For the list from GeneCards, there were 144 genes with a relevance score ≥12 from an initial number of 5,735 genes. On the other hand, there were 30 insulin resistance genes downloaded from DisGeNET with disease Unified Medical Language System (UMLS) concept unique identifier (CUI) C0021655 for insulin resistance. The intersection of these gene sets with the set of *M. oleifera* targets produced a list of nine relevant proteins (Figure 2). The protein targets relevant to insulin resistance targeted by *M. oleifera* phytochemicals, along with their subcellular location from Uniprot and the PDB files used for molecular docking, are listed in Table 1. Protein-protein interactions of the relevant insulin resistance targets were retrieved from the STRING database (Supplementary Table S5). Based on calculated k-means in STRING, these targets can be grouped into two clusters: 1) Regulation of lipid storage, and 2) Phosphatidylinositol 3-kinase complex, class 1, and insulin receptor complex.

Overall, there were a total of 170 phytochemical-protein interactions between 85 phytochemicals and nine insulin resistance targets. The complete phytochemical-protein interaction network is shown in Supplementary Figure S1.

Degree centrality refers to the number of direct connections or edges to the node. Betweenness centrality is interpreted as the degree to which a node acts as a bridge or bottleneck and facilitates communication between different regions. Closeness centrality may be interpreted as the degree to which a node is efficiently connected to the entire network and rapidly propagates its effects.

Considering the complete network which also considers probable nonbinders, PTPN1 is the most important node in three network centrality measures: degree, betweenness centrality, and closeness centrality (Supplementary Table S6). However, most predicted phytochemical interactions to PTPN1 are relatively weak, i.e., the binding affinities, while negative, have low magnitude. Nevertheless, the inhibition of PTPN1 by *M. oleifera* extract has already been previously demonstrated. In a study that involved the crude methanol extract of 11 different plants, *M. oleifera* extract ranked fourth with 41.2% inhibition, behind *Senna nigricans*, *Azadirachta indica*, *Arachis hypogaea*, and *Acacia nilotica* (Saidu et al., 2016). PTPN1 was also previously identified as a key target *via* network pharmacology analysis (Huang et al., 2020).

Molecular docking with Autodock Vina was used to evaluate the phytochemical-protein interactions. This is because the calculated probability using Swiss Target Prediction is only based on shape similarity to known ligands of the target and does not provide information on the specific interactions between the phytochemical and the target. The binding affinity calculated from docking can also serve to estimate the strength of interaction between them. However, predicted binding affinity can diverge from the actual experimental value because of deficiencies of the scoring function in predicting bond strength, solvation effects, entropy, or polarization (Spassov, 2024). It is typically assumed that more negative binding energies imply more favorable binding of the target with the ligand. While this is generally true, a predicted negative binding affinity does not necessarily translate to actual inhibition or activation.

Hence, we have endeavoured to compare the binding affinity of the phytochemical-protein interactions with the binding affinities of: (a) binders, i.e., annotated drugs and other molecules with known interaction with each target, taken from DrugBank, BindingDB, redocked ligands for each target from PDB, and phytochemicals with Swiss Target Prediction predicted probability = 1; and (b) nonbinders which are not expected to interact with each target. Nonbinders used were glycerol, phenylacetonitrile and cyclohexane, glyphosate, dicamba, paraquat, glufosinate, and decoys generated by DUDE-Z for each target from the SMILES of annotated ligands or binders. These nonbinders should not target insulin resistance proteins based on Swiss Target Prediction results. At least 20 ligands were chosen for each category (binders and nonbinders) and protein target (Youden, 1950). The binders and nonbinders used for each target are listed in Supplementary Tables S7 and S8.

Decoys that represent the characteristics of the library to be docked are commonly used to evaluate and optimize docking library screens. DUDE-Z (Stein et al., 2021) is an optimized version of the property-matched Directory of Useful Decoys–Enhanced (DUD-E) set (Mysinger et al., 2012) that builds all protonation states of submitted ligands at physiological pH to overcome unintended biases leading to false enrichment. Decoys are matched to ligands based on molecular weight, water-octanol partition coefficient (log *P*), number of rotatable bonds, number of hydrogen bond donors and acceptors, and net charge. After decoys are retrieved from the ZINC website for each protomer, these are sorted by Tanimoto calculations and molecular weight iteratively to discard decoys that are too similar topologically to any submitted ligand.

In the current study, receiver operating characteristic (ROC) curves were used to determine if binding affinities from docking can correctly classify binders and nonbinders. ROC was developed originally as a method in radio communication to distinguish between actual radio signals and noise, but is now applied in various fields such as psychology, radiology, epidemiology, finance, weather forecasting, social sciences, medicine, bioinformatics, data mining and machine learning (Tan, 2009; Robin et al., 2011). An ROC plot displays the performance of a binary classification method as a plot of sensitivity *versus* specificity across the range of all possible values. Sensitivity is also known as the true positive rate (TPR) and is defined as the proportion of all correctly classified positive observations. Specificity is defined as the proportion of all correctly classified negative observations and is related to the false positive rate (FPR) as (1 – FPR). A perfectly accurate classification method would yield an area-under-curve (AUC) of 1, whereas an AUC value of 0.5 indicates that the classification method has no predictive power and is only the result of random chance. In this study, Youden’s J-statistic was used to establish the optimal threshold binding affinity for classifying phytochemicals as probable binders or probable nonbinders. Youden’s J-statistic maximizes the vertical distance between the ROC curve and the diagonal which represents random chance where AUC is 0.5 (Sonego et al., 2008; Robin et al., 2011; Nahm, 2022; Chang and Newman, 2024).

**FIGURE 3:**
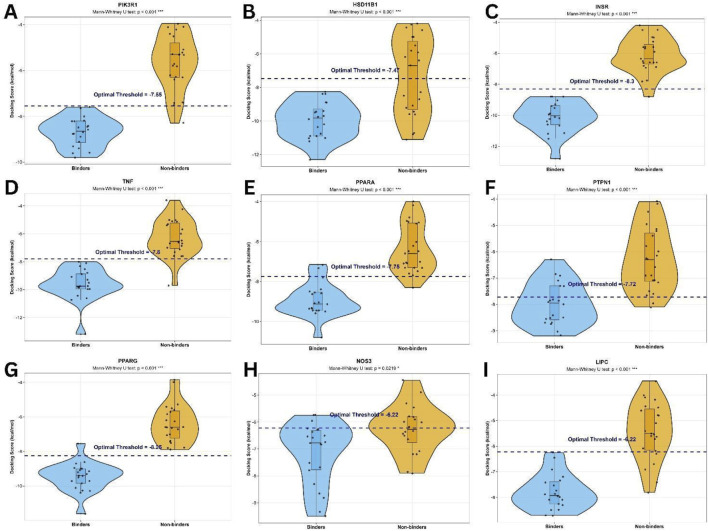
Distribution plots of docking scores of binders (blue) and nonbinders/decoys (yellow) for each protein target: **(A)** PIK3R1 **(B)** HSD11B1, **(C)** INSR, **(D)** TNF-α, **(E)** PPARα, **(F)** PTPN1, **(G)** PPARγ, **(H)** eNOS, **(I)** LIPC. Images created using RStudio with ggplot2.

The distribution plots of docking scores of binders and nonbinders for each target and the calculated thresholds using the pROC plugin in R are shown in Figure 3. Most area-under-curve (AUC) values indicate good (AUC ≥0.8) or excellent (AUC ≥0.9) discrimination between binders and nonbinders, except for NOS3/eNOS with moderate discrimination (AUC ≥0.7) (Supplementary Figure S2) (In the case of eNOS, predicted binding affinity of its probable binders are well below the calculated threshold.) Furthermore, Mann-Whitney tests (*in lieu* of t-test, as the distributions are non-normal) all have very significant (<0.001) or significant (<0.05) p-values indicating a definite difference between mean docking scores of binders *versus* nonbinders. Cohen’s d values are −0.842 for eNOS, and between −1.401 and −3.652 for the other targets, indicating good to excellent separation between groups. The calculated enrichment factors at 20% of 1.59–2.25 indicate successful ranking of most of the actual binders. Detailed statistical analyses of docking scores of binders and nonbinders are provided in Supplementary Table S9. Phytochemical-protein target interactions were then classified either as probable binders or probable nonbinders if the average docking score or binding affinity was below or above the optimal threshold value, respectively, for best accuracy (Supplementary Table S10).

## 4 Discussion

### 4.1 Phytochemical set and target prediction

Huang et al. (2020) also employed a network pharmacology approach to explore the mechanism of anti-insulin resistance effects of *M. oleifera* seeds, but we would like to point out a few differences from the approach used in this study. These differences underscore the need for further standardization of the methodology of network pharmacology specifically in the prediction services and databases used in the approach.

First, they used an initial set of 46 phytochemicals, predominantly isothiocyanates and benzylamines, with the knowledge that these phytochemical classes are known to exhibit various activities. Some isothiocyanates and benzylamines were included despite not fulfilling the criteria for oral bioavailability and drug-likeness that were enforced for other chemical constituents. Conversely, we did not wish to limit the phytochemical list in this study to only those with known activities. After filtering the initial number of 290 *M. oleifera* phytochemicals according to ADME criteria, 168 phytochemicals of various phytochemical classes were submitted to Swiss Target Prediction for reverse screening of possible targets.

Second, they used several databases for target prediction including Swiss Target Prediction, Similarity Ensemble Approach, PharmMapper, and STITCH. On the other hand, this study employed Swiss Target Prediction only for reverse screening. Despite using only one database for target prediction, there were only a handful of *M. oleifera* targets that were not among the target list of Huang et al. (2020), namely: HGF, PCK1, EIF4E, CXCR4, G6PC, and CREB1. These targets are of relatively low importance in the context of insulin resistance. Among this list, the target with the highest relevance score in GeneCards is G6PC, with a score of 10.63, below the cutoff used for this study. This is supported by an assessment of nine target prediction web services which asserted that Swiss Target Prediction and Similarity Ensemble Approach may be used as primary selection methods, and that Swiss Target Prediction produced the most reliable predictions while enriching more targets (Ji et al., 2023).

In contrast with other previous network pharmacology studies, the list of genes relevant to insulin resistance, the disease of interest in this study, is relatively short with only nine genes. The primary difference is the additional screening using the relevant gene list from DisGeNET under Unified Medical Language System (UMLS) concept unique identifier (CUI) C0021655 for insulin resistance consisting of only 30 genes initially. This is not necessarily a shortcoming but reflects the differences in curation methods of various databases. Both GeneCards and DisGeNET employ curated databases Uniprot and Orphanet as data sources, however, each also employs data sources that are not used by the other. For example, according to our Swiss Target Prediction results, matrix metalloproteinases MMP2 and MMP9 are targeted by 67 and 63 phytochemicals, respectively. Research is showing that MMPs indeed play a role in cancer, inflammation, and other metabolic diseases (Molière et al., 2023), but, currently, involvement in insulin resistance in particular is not listed for these proteins in the Uniprot database. This study used the intersection of the sets of genes from GeneCards and DisGeNET to come up with a more concise list of targets that can be studied with more depth using statistical methods in the next steps.

### 4.2 Network visualization enhancement with subcellular location and ADME parameters

**FIGURE 4:**
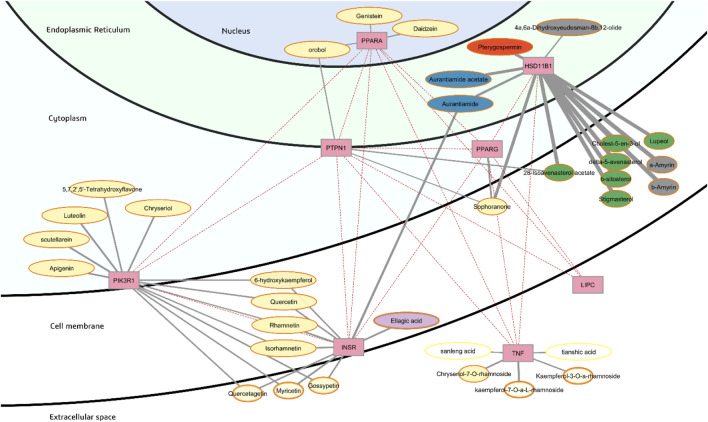
Annotated phytochemical-protein interaction network considering probable binders only with their ADME parameters and subcellular location of targets. Image created using Cytoscape.

After classification of predicted binding affinities and removal of probable nonbinders, the initial number of 170 interactions between phytochemicals and targets relevant to insulin resistance were narrowed down to 52 possible interactions between 40 phytochemicals with 8 hub targets in 2 clusters. The plausible targets are a) HSD11B1, TNF-α, PPARα, PPARγ, and eNOS in the cluster for regulation of lipid storage, and b) PIK3R1, INSR, and PTPN1 in the cluster involving the metabolic PI3K pathway. The simplified network showing only probable binders is presented in Figure 4. There were no probable binders predicted to interact with LIPC. All targets except for LIPC are of some importance considering the network centrality measures (Table 2).

**TABLE 2:** Network centrality measures considering probable binders only.

Target	Degree		Betweenness		Closeness	
	Value	Rank	Value	Rank	Value	Rank
PIK3R1	17	1	0.307	2	0.439	6
HSD11B1	16	2	0.433	1	0.448	5
INSR	14	3	0.267	3	0.480	3
TNF	10	4	0.265	4	0.475	4
PPARA	10	4	0.214	5	0.495	2
PTPN1	9	5	0.153	7	0.480	3
PPARG	8	6	0.177	6	0.511	1
NOS3	5	7	0.084	8	0.402	7
LIPC	3	8	0	-	0.376	8

**FIGURE 5:**
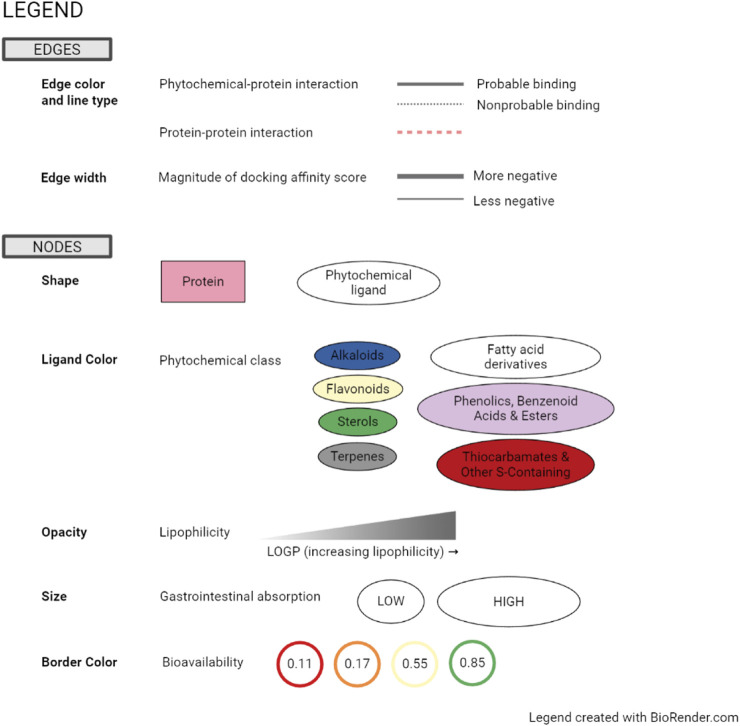
Legend for network visualizations incorporating subcellular location, ADME parameters, and probability of binding.

Organization of the targets according to subcellular location is a useful aspect of the visualization as it facilitates assessment of whether phytochemicals will be able to reach the intended targets. Likewise, additional information such as ADME parameters and predicted binding affinity are highlighted and made more meaningful by this style of visualization (Briones et al., 2021). The detailed legend is presented in Figure 5.

In this study, the phytochemicals that are probable binders also all have moderate bioavailability, represented as orange or yellow borders in the visualization. Lipophilicity, represented as opacity of phytochemical nodes, is important to assess whether the subcellular environment will be favorable and if the phytochemical will be able to cross the cell membrane. In SwissADME, consensus log *P* is the arithmetic mean of the results from five methods to compute the logarithm of the partition coefficient (log *P*), defined as follows:

$$\log\;P=\frac{concentration\;of\;the\;solute\;in\;octanol}{concentration\;of\;the\;solute\;in\;water}$$

More lipophilic substances will carry greater values of log *P*, and a negative value suggests that the solute favours an aqueous environment and is not likely to cross the cell membrane. This is a concern for all phytochemicals in this study except for a few that are predicted to target secreted proteins TNF-α and LIPC. The optimal log *P* to cross biological membranes is generally considered to be −1.0 to 2.0 (Zou et al., 2022).

Predictions in SwissADME for passive human gastrointestinal absorption are based on the Brain Or IntestinaL EstimateD permeation (BOILED-Egg) model which plots Wildman and Crippen log *P* (WLOGP) *versus* the TPSA (Daina and Zoete, 2016). This model is based on human intestinal absorption data of a library of well and poorly absorbed small molecules. The points that fall outside of the plot that resembles a boiled egg are considered to have low gastrointestinal absorption. In this study, predicted gastrointestinal absorption of phytochemicals is represented as the size of the nodes.

Bioavailability score in SwissADME is assigned as the probability that a compound will have sufficient bioavailability (>10%) in rat. It was based on a dataset of rat bioavailability data and assigned based on the charge state at pH 6–7. For anions, bioavailability score is governed by polar surface area, whereas for other compounds, Lipinski’s rule-of-five for drug-likeness is the main predictor (Martin, 2005). In this study, bioavailability score is represented as the border color of the phytochemical nodes.

In this study, all phytochemicals that are probable binders have positive consensus log *P* values. However, the flavonoid sophoranone, the sterols cholest-5-en-3-ol, stigmasterol, β-sitosterol, Δ^5^-avenasterol, lupeol, and 28-isoavenasterol acetate, and the triterpenes α-amyrin and β-amyrin are too lipophilic (consensus log *P* > 5). Although it is possible for these phytochemicals to be absorbed into the cell membrane, they will also be likely retained there and may not be able to interact with the targets HSD11B1 and PTPN1. Their high predicted lipophilicity is anticipated to also contribute to their low gastrointestinal absorption.

The flavonoids quercetagetin, myricetin, gossypetin, chryseriol-7-O-rhamnoside, kaempferol-7-O-rhamnoside, and kaempferol-3-O-rhamnoside are also predicted to have low gastrointestinal absorption based on WLOGP and TPSA. For myricetin, the calculated oral bioavailability agrees with previous experimental data, as only around 10% of myricetin was absorbed in rat plasma after oral administration (Park et al., 2016). The predicted targets of the flavonoids mentioned are mostly in the extracellular space (TNF-α) and on the cell surface (INSR). Quercetagetin, myricetin and gossypetin are also predicted to target PIK3R1 but would less likely be able to do so because of their low estimated oral bioavailability.

Hence, it would be reasonable to remove from our consideration phytochemicals with low predicted gastrointestinal absorption and oral bioavailability, and those with that have consensus log *P* that are too high and too low, especially if their targets are within the cell and not in the extracellular space. Phytochemicals that are probable binders to insulin resistance targets and with high predicted gastrointestinal absorption are listed in Table 3. Most of these are categorized as flavonoids; these along with ellagic acid are predicted to target the cluster of INSR and PIK3R1 involved in the metabolic PI3K pathway. The active site of INSR is within the plasma membrane, while PIK3R1 is located just below the inner leaflet of the plasma membrane. Ellagic acid, a phenolic acid, is predicted to target INSR only. The predicted high gastrointestinal absorption and lipophilicity of flavonoids and the phenolic ellagic acid are with consensus log *P* of around 1.0 to 2.2 makes it likely that they will be able to cross the plasma membrane and reach the targets within the cell. The flavonols robinetin, rhamnetin, isorhamnetin, quercetin and 6-hydroxykaempferol are predicted to target both the insulin receptor INSR, and PIK3R1, the regulatory subunit of PI3K. The flavones luteolin, scutellarein, apigenin, chryseriol, 2′,5,5′,7-tetrahydroxyflavone and 2′,3,5,7-tetrahydroxyflavone are predicted to target PIK3R1 only.

**TABLE 3:** *Moringa oleifera* phytochemicals with high gastrointestinal absorption that are probable binders to insulin resistance targets.

Phytochemical class	Phytochemical name	Consensus log *P*	Target	Predicting binding affinity (kcal/mol)
Steroid	Boldione	3.33	HSD11B1	−11.3
Peptide derivative	Aurantiamide acetate	3.89	HSD11B1	−10.2
	Aurantiamide	3.37	HSD11B1	−9.7
	Aurantiamide	3.37	INSR	−9.3
Thiocarbamate	Pterygospermin	3.75	HSD11B1	−10.0
	O-Ethyl [(3,4-dihydroxyphenyl)methyl]carbamothioate	1.58	NOS3	−7.2
	O-Methyl N-[(4-hydroxyphenyl)methyl]carbamothioate	1.59	NOS3	−6.9
Phenolic acid	Ellagic acid	1.00	INSR	−8.8
Sesquiterpene	4α,6α-Dihydroxyeudesman-8β,12-olide	1.77	HSD11B1	−8.2
Fatty acid derivative	Sanleng acid	3.02	TNF	−8.1
	Tianshic acid	3.08	TNF	−8.1
Flavonoid	Luteolin	1.73	PIK3R1	−8.9
	Scutellarein	1.81	PIK3R1	−8.9
	Apigenin	2.11	PIK3R1	−8.7
	Chryseriol	2.18	PIK3R1	−8.7
	2′,5,5′,7-Tetrahydroxyflavone	1.74	PIK3R1	−8.6
	Robinetin	1.12	PIK3R1	−8.5
	Quercetin	1.23	PIK3R1	−8.4
	6-Hydroxykaempferol	1.28	PIK3R1	−8.4
	Isorhamnetin	1.65	PIK3R1	−8.2
	Rhamnetin	1.63	PIK3R1	−8.1
	2′,3,5,7-Tetrahydroxyflavone	1.61	PIK3R1	−7.8
	Robinetin	1.12	INSR	−8.7
	Rhamnetin	1.63	INSR	−8.7
	Isorhamnetin	1.65	INSR	−8.5
	Quercetin	1.23	INSR	−8.5
	6-Hydroxykaempferol	1.28	INSR	−8.4
	Daidzein	2.24	PPARA	−8.1
	Orobol	1.73	PPARA	−7.9
	Genistein	2.04	PPARA	−7.8
	Orobol	1.73	PTPN1	−8.0

Meanwhile, the isoflavones daidzein, orobol, and genistein are predicted to target PPARA located inside the nucleus. Their predicted consensus log *P* of 1.73–2.24 makes it probable that these would be able to cross the cell membrane. Entry into the nucleus is *via* facilitated diffusion through nuclear pore complexes, and the nuclear envelope is considered freely permeable to small molecules of 5,000 Da or less (Alberts et al., 2002). Aside from PPARA, orobol is also predicted to target PTPN1 which is on the surface of the endoplasmic reticulum facing the cytosol.

The predicted high gastrointestinal absorption of isoflavones is in accord with experimental studies. For example, daidzein is absorbed by passive transport in the small intestine of rats, with higher absorption in the distal region (Foti et al., 2006). Genistein is also known to be efficiently absorbed from the intestines although much of it is metabolized in the liver and excreted (Sfakianos et al., 1997).

Modified fatty acids sanleng acid and tianshic acid are predicted to have probable binding with TNF-α which is in the extracellular space. Sanleng acid and tianshic acid are foreseen to be very lipophilic, with estimated consensus log *P* of around 3.0. Although the target TNF-α is in the aqueous extracellular space, the lipophilicity of fatty acids is not expected to be a hindrance as fatty acids are transported in the blood primarily through incorporation into lipoproteins or binding to albumin.

HSD11B1 is anticipated to be targeted by different phytochemical classes, namely, the steroid boldione, peptide derivatives aurantiamide acetate and aurantiamide, the sesquiterpenoid 4α,6α-dihydroxyeudesman-8β,12-olide, and the thiocarbamate pterygospermin. Aurantiamide additionally is also predicted to have probable binding with INSR. These phytochemicals have high calculated gastrointestinal absorption, and indeed, aurantiamide acetate and aurantiamide have been found to be rapidly absorbed after oral administration in rats (Chen et al., 2016). Except for 4α,6α-dihydroxyeudesman-8β,12-olide which has a computed consensus log *P* of 1.77, these compounds are expected to be quite lipophilic, with consensus log *P* in the range of 3.33–3.89, appropriate for the location of the target HSD11B1 which is in the inner leaflet of the endoplasmic reticulum membrane.

Probable binders of eNOS are the thiocarbamates O-methyl-N-[(4-hydroxyphenyl)methyl] carbamothioate and O-ethyl [(3,4-dihydroxyphenyl)methyl] carbamothioate, with favorable consensus log *P* of 1.59 and 1.58 respectively for the putative target which is for the most part localized to plasma membrane. This target has no transmembrane domain, but post-translational myristoylation or palmitolylation near the N-terminus is thought to stabilize membrane association or redistribution to lipid domains (Shu et al., 2015).

### 4.3 Probable targets in the regulation of lipid storage cluster

#### 4.3.1 11-beta hydroxysteroid dehydrogenase 1 (HSD11B1)

**FIGURE 6:**
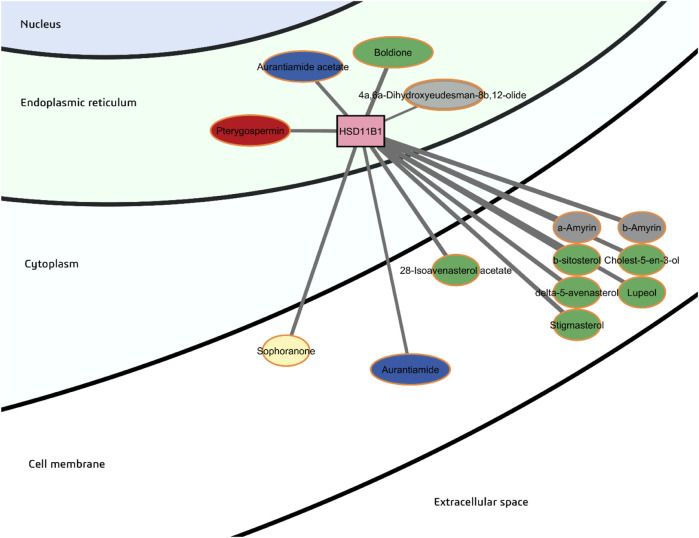
Probable binders of 11-beta-hydroxysteroid dehydrogenase 1 (HSD11B1).

HSD11B1 or 11-beta-hydroxysteroid dehydrogenase 1 is estimated to be the second most important node in the network in terms of degree and most important in terms of betweenness centrality (Table 2). HSD11B1 functions to convert cortisone, an inactive glucocorticoid, to cortisol, the active form, within insulin-sensitive tissues. In the network shown in Figure 6, it is apparent that HSD11B1 may exhibit strong probable binding with sterols and triterpenes. This is not surprising given the structural similarity of these phytochemicals with cortisol. These sterols and triterpenes along with the flavonoid sophoranone have relatively high predicted consensus log *P* > 6 and low predicted GI absorption. Although these could possibly cross the bilipid layer of the cell membrane by passive diffusion, they will most likely be retained there. Aurantiamide acetate, aurantiamide, boldione, and pterygospermin, with consensus log *P* in the range of 3.33–3.89, are predicted to be more drug-like as they are lipophilic but to a lesser magnitude as compared to sterols and triterpenes. The sesquiterpenoid 4a,6a-dihydroxyeudesman-8b,12-olide has the most favorable calculated consensus log *P* at 1.77.

The location of HSD11B1 is within the smooth endoplasmic reticulum (SER), so-called as it lacks bound ribosomes. The SER functions for lipid and steroid synthesis and is prominent in cells that specialize in lipid metabolism, mainly adipocytes and hepatocytes. HSD11B1 is oriented in the inner leaflet of the SER membrane, with its catalytic domain containing the C-terminus facing the ER lumen (Odermatt et al., 1999). The catalytic region with a triad of Ser, Tyr and Lys residues is closely associated with hexose-6-phosphate dehydrogenase (H6PDH) in the lumen to ensure efficient regeneration of the coenzyme NADPH from NADP^+^ *via* the first two steps of the pentose phosphate pathway (Gomez-Sanchez and Gomez-Sanchez, 2021).

Increased expression of HSD11B1 has been associated with obesity and insulin resistance (Dujic et al., 2012). HSD11B1 is highly relevant in the progression of the disease because cortisol drives lipid deposition in the liver and fat cells, resulting in dysfunction and insulin resistance (Abulizi et al., 2019). Besides redox interconversion between cortisone and cortisol, HSD11B1 may also play a role in the formation of oxidized cholesterol metabolites such as 7-oxycholesterol which is abundant in atherosclerotic plaques (Odermatt et al., 2006). It has also been shown to catalyze the conversion of androstenedione to testosterone, activation of exogenous glucocorticoids such as prednisolone and betamethasone, and detoxification of xenobiotics such as metyrapone and 4-nitrobenzaldehyde (Oestlund et al., 2024).

It has been suggested that the large hydrophobic surface at the C-terminus of HSD11B1 allows funneling of the substrate from the lipid bilayer into the active site cleft (Chapman et al., 2013). Visualization of the interactions between HSD11B1 and aurantiamide acetate (a peptide derivative) as well as the sesquiterpenoid 4α,6α-dihydroxyeudesman-8β,12-olide in the docked structure show important predicted hydrophobic interactions within this pocket (Supplementary Figure S3) which may block entrance of the substrate into the active site. Key residues of the enzyme include the catalytic residues Ser170, Tyr183, and Lys187, Tyr177, which are critical for cortisone binding, and the Ser228-Pro237 loop which functions for ligand recognition (Favia et al., 2011).

#### 4.3.2 Tumor necrosis factor alpha (TNF-α)

**FIGURE 7:**
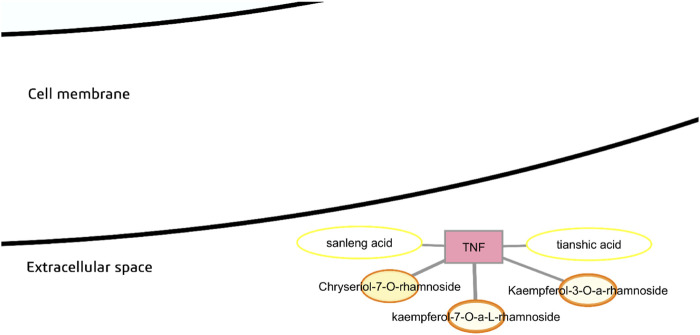
Probable binders of tumor necrosis factor α (TNF-α).

*Moringa oleifera* phytochemicals predicted to have probable binding with tumor necrosis factor α (TNF-α) are fatty acid derivatives tianshic acid and sanleng acid, and glycosylated flavonoids kaempferol-7-O-rhamnoside, kaempferol-3-O-rhamnoside, and chryseriol-7-O-rhamnoside (Figure 7). Among these, fatty acid derivatives have higher predicted bioavailability and gastrointestinal absorption than glycosylated flavonoids. In the best docking poses, both tianshic acid and sanleng acid demonstrate possible hydrophobic interactions with Tyr195 and other nonpolar residues in the binding pocket (Supplementary Figure S4).

TNF-α is a proinflammatory cytokine secreted by adipocytes and immune cells that is well-associated with chronic inflammation and impairment of insulin signaling (Mohallem and Aryal, 2020). Although regulation of TNF-α is typically focused on reducing proinflammatory effects, studies have shown that TNF-α may also be important in mediating repair mechanisms such as myelin repair after CNS injury (Raffaele et al., 2020). In the obese state, however, there is an excessive infiltration of adipose tissue by immune cells; macrophages constitute up to 40% of all adipose tissue cells (Zatterale et al., 2020). The inflammatory response results in increased secretion of c-Jun N-terminal kinase (JNK) and nuclear factor kappa-light-chain-enhancer of activated B cells (NF-κB). Additionally, TNF-α reduces the expression of glucose transporter type 4 (GLUT4) in insulin-sensitive cells and also induces serine phosphorylation (rather than tyrosine phosphorylation) of insulin receptor substrate-1 (IRS-1) thereby inhibiting peripheral insulin action (Alzamil, 2020).

TNF-α is produced initially as a transmembrane protein by immune cells and cleaved by TNF-α converting enzyme or metalloprotease domain 17 (TACE/ADAM17), generating the soluble form of TNF-α which is secreted into the extracellular space (Raffaele et al., 2020). The active form of TNF-α has a trimeric structure which is key for it to trigger intracellular signaling (Smith and Baglioni, 1987). The active site of TNF-α is in the interface of the trimer structure where a cognate receptor, TNFR1 or TNFR2, can engage. One strategy for TNF-α inhibition is the use of biologics, such as the monoclonal antibodies infliximab and adalimumab, that bind to TNF-α and block engagement with the receptor. Another mechanism is disruption of the TNF-α trimer using small molecule inhibitors *via* allosteric regulation (Chédotal et al., 2023). These allosteric inhibitors typically form hydrogen bonding or pi-stacking interactions with specific tyrosine residues (Tyr119, Tyr59, Tyr151, Tyr227, Tyr135, Tyr195) and hydrophobic interactions with nonpolar residues in the trimeric interface region that prevent effective binding of TNFR (O’Connell et al., 2019; Xiao et al., 2020).

#### 4.3.3 Proliferator-activated receptor alpha (PPARα)

**FIGURE 8:**
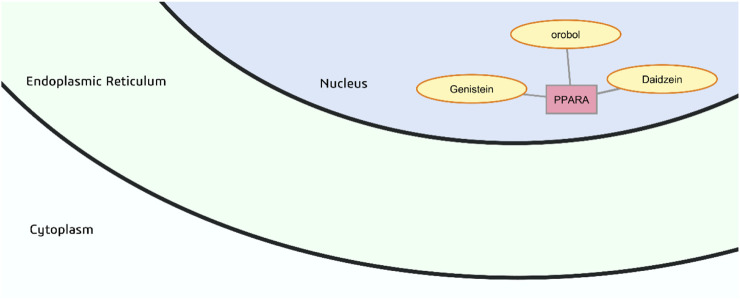
Probable binders of peroxisome proliferator-activated receptor α (PPARA).

The isoflavones orobol, genistein and daidzein are predicted to have probable binding with PPARα (Figure 8). Their predicted gastrointestinal absorption and consensus log *P* of 1.73–2.24 are favorable for crossing the cell membrane. Once accomplished, entry into the nucleus through facilitated diffusion through nuclear pore complexes will not be impeded as the nuclear envelope is considered freely permeable to small molecules of 5,000 Da or less (Alberts et al., 2002).

PPARα is the pharmaceutical target of a class of drugs called fibrates used for the treatment of dyslipidemia. It is located in the nucleus and a key regulator of lipid metabolism, particularly of genes that are involved in fatty acid oxidation and ketone body production such as fatty acid transport proteins, long-chain acyl-CoA synthetase, acyl-CoA oxidase, carnitine palmitoyl transferase I, medium-chain acyl-CoA dehydrogenase, and hydroxymethylglutaryl-CoA synthase. By inducing expression of pyruvate dehydrogenase kinase 4 which phosphorylates and inactivates pyruvate dehydrogenase, PPARα also has a glucose sparing effect. Activation of PPARα in various models of insulin resistance improves insulin sensitivity by increasing both peroxisomal and mitochondrial oxidation of fatty acids and reducing lipotoxicity (Ferré, 2004). A preclinical study using C2C12 myoblasts demonstrated that treatment with *M. oleifera* leaf extract positively modulated glucose and free fatty acid consumption, as well as protein expression of PPARα along with SIRT1 (Duranti et al., 2021).

The crystal structure of indeglitazar, an antidiabetic agent that can activate all PPAR isoforms (α, γ, and δ), with PPARα shows interactions with key aromatic residues Tyr314, Tyr464, Phe318, and His440 in the ligand binding domain (Artis et al., 2009), which are also notable in the docked structures of PPARα with flavonoids such as daidzein (Supplementary Figure S5).

#### 4.3.4 Proliferator-activated receptor gamma (PPARγ)

**FIGURE 9:**
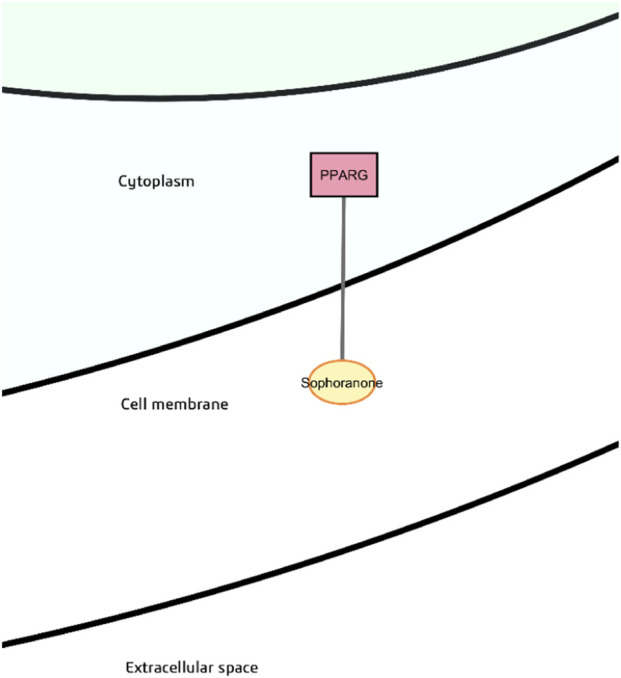
Probable binder of peroxisome proliferator-activated receptor γ (PPARG).

In this study, PPARγ is predicted to have probable binding with sophoranone, calculated to have very low polarity with consensus log *P* of 6.36, and low gastrointestinal absorption (Figure 9). Although there were no probable binders with PPARγ with predicted high gastrointestinal absorption from among the *M. oleifera* phytochemicals, it ranked well with other targets in terms of closeness centrality because of protein-protein interactions (Table 2). This implies that PPARγ is efficiently connected to the network, and it can be supposed that there would be secondary influence on PPARγ if the other targets are acted upon.

PPARγ is a ligand-activated transcription factor in the same family as PPARα. It is highly expressed in adipose tissue, lower intestine, and immune cells. When present in the cytoplasm, PPARγ is sequestered by phosphorylation, but once activated by a ligand, it can translocate to the nucleus and dimerize with retinoic acid X receptor (RXR) for the transcription of various genes for lipid and glucose homeostasis (Govindarajulu et al., 2018). PPARγ activation promotes lipid storage in white adipocytes in the well-fed state, to prevent lipid accumulation in liver and skeletal muscle, as well as uptake of glucose in skeletal muscle and suppression of hepatic gluconeogenesis. In immune cells, activation of PPARγ leads to secretion of adiponectin and induction of an anti-inflammatory phenotype (Basak et al., 2024; Ferré, 2004). Thiazolidinedione (TZD) drugs, such as rosiglitazone and pioglitazone, used for the treatment of insulin resistance are potent agonists of PPARG. TZD drugs form hydrogen bonds with several residues in the AF-2 pocket of the ligand-binding domain of PPARγ and induce structural changes in the protein. The AF-2 pocket is composed of many polar residues such as Cys285, Ser289, His323, Tyr327, His449, and Tyr473. There are also numerous nonpolar residues in the central region of the ligand-binding pocket such as Leu330, Leu339, Leu353, and Met364 (Gelin et al., 2015; Lee et al., 2017). Sophoranone, in its best docking pose with PPARγ, exhibited possible hydrophobic interactions with Cys285, Leu330 and Met364 (Supplementary Figure S6).

#### 4.3.5 Endothelial nitric oxide synthase (eNOS, NOS3)

Endothelial nitric oxide synthase (eNOS, NOS3) is primarily produced in endothelial cells that line the interior of blood vessels. It functions primarily for the synthesis of nitric oxide (NO) that acts as a vasodilator and anti-inflammatory mediator in these cells.

**FIGURE 10:**
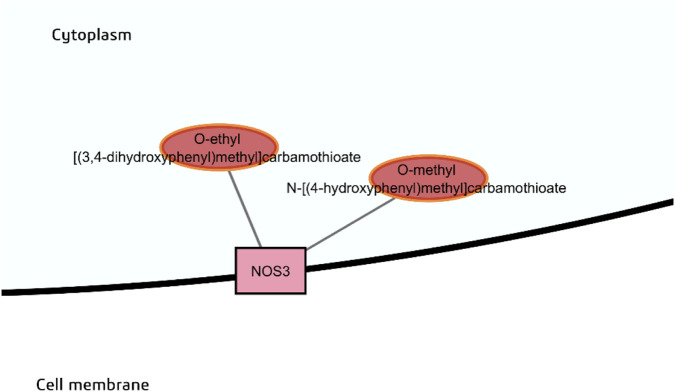
Probable binders of endothelial nitric oxide synthase (eNOS).

Thiocarbamate aglycones O-methyl-N-[(4-hydroxyphenyl)methyl] carbamothioate (the aglycone of niazinin A) and O-ethyl-[(3,4-dihydroxyphenyl)methyl] carbamothioate were predicted to have probable binding with eNOS (Figure 10). These were both predicted to have moderate bioavailability, high GI absorption and a favorable consensus log *P* of 1.58 and 1.61, respectively, to be capable of crossing the cell membrane. Their rhamnosylated counterparts were computed to be more polar, with consensus log *P* of 0.42 and 0.47, respectively, and the latter was predicted to have low GI absorption.

Endothelial NOS is a homodimeric protein with each subunit separated into an oxygenase domain (residues 98–486), where NO synthesis occurs, and a reductase domain (residues 756–1002) that are linked by a calmodulin-binding domain (residues 491–510). Only the calcium-bound calmodulin can bind to this domain, hence activity of eNOS is tied to intracellular Ca^2+^ concentration. The enzyme also requires the presence of several cofactors, namely, NADPH, FAD and FMN in the reductase domain, and tetrahydrobiopterin (BH_4_), iron-containing heme, and Zn^2+^ in the oxygenase domain. Electrons are transferred sequentially thus: NADPH -> FAD -> FMN -> Fe^3+^. Reduced heme then catalyzes the synthesis of NO from substrates L-arginine and molecular oxygen, forming citrulline as a by-product (Shu et al., 2015). BH_4_ provides a second electron for the oxidation of arginine and binds near the interface of the subunits, stabilizing the dimeric structure (Schaffer et al., 2012). Zinc is structurally required as a coordination center for several cysteine residues but does not participate in electron transfer.

Aside from calmodulin, eNOS can bind to other regulatory partners at other sites within the oxygenase domain. For this target, only partial structures comprised mostly of the oxygenase domain that binds NOS-interacting protein (NOSIP) are currently available in the Protein Data Bank. NOSIP decreases NO production by trafficking eNOS from the membrane into intracellular sites for ubiquitination (Dedio et al., 2001). For this reason, the box used for docking was centered around residues 366–486 of the NOSIP-binding domain. Inhibition of eNOS is not desirable in the context of insulin resistance, so the ligand L-arginine, which is the substrate, and other co-factors were not removed prior to docking.

In their best docked poses, the thiocarbamate aglycones O-methyl-N-[(4-hydroxyphenyl)methyl] carbamothioate and O-ethyl-[(3,4-dihydroxyphenyl)methyl] carbamothioate were predicted to form hydrogen-bonding and hydrophobic interactions with residues in the NOSIP-binding domain, so it is possible that these thiocarbamates may disrupt binding of NOSIP and hence prevent sequestration of eNOS. It was also interesting to note the position of the thiocarbamates in the predicted structures. The thiocarbamate would be situated between the heme cofactor in the oxygenase domain and the reductase domain of the other subunit. A cysteine residue is just adjacent to the sulfur-containing thiocarbamate (Supplementary Figure S7). We speculate that that this might aid the electron transfer reaction or quenching of reactive oxygen species.

Although the effect of moringa phytochemicals on eNOS has not been directly observed, previous studies have shown that carbamate and thiocarbamate glycosides of *M. oleifera* possess hypotensive activity (Faizi et al., 1994). Moreover, *M. oleifera* leaf extract has been demonstrated to alleviate high blood pressure and oxidative stress in N^ω^-nitro-L-arginine-methyl ester (L-NAME)-induced hypertensive rats (Aekthammarat et al., 2019). L-NAME is a competitive inhibitor of L-arginine for the NOS isoforms. This study illustrates that the aglycones of thiocarbamate glycosides of *M. oleifera* may be the actual bioactive forms of the compounds that may specifically target eNOS. Removal of the carbohydrate moiety may also serve to improve GI absorption as well as lipophilicity to enable entry across the cell membrane of endothelial cells.

#### 4.3.6 LIPC

Hepatic lipase (LIPC) is a secreted protein from the liver that catalyzes the hydrolysis of triglycerides and phospholipids present in circulating plasma lipoproteins. LIPC was ranked lowest in three network centrality measures, and there were no phytochemicals with predicted probable binding, implying that it is of the least importance among all the relevant targets of *M. oleifera* phytochemicals related to insulin resistance.

### 4.4 Probable targets in the metabolic PI3K pathway cluster

Insulin is a peptide hormone secreted by beta cells of the pancreatic islets. It is the main anabolic hormone of the body and promotes the absorption of glucose from the blood into insulin-sensitive cells, namely, hepatic, adipose, and skeletal muscle cells. There are two main branches of insulin signaling: 1) the mitogenic Ras MAPK pathway, which promotes cell proliferation, differentiation, motility, and survival, and 2) the metabolic PI3K-AKT pathway, which promotes glucose uptake, glycogen synthesis, lipogenesis, gluconeogenesis, and lipolysis. The PI3K signal cascade of the latter branch of insulin signaling results in glucose transporter 4 (GLUT4) translocation from intracellular vesicles to the plasma membrane for glucose to enter the cell (Olivares-Reyes et al., 2009; Reddy et al., 2010; Jung and Choi, 2014; Świderska et al., 2018). A cluster of *M. oleifera* targets, namely, PIK3R1, INSR, and PTPN1, are involved in the latter.

#### 4.4.1 PI3-kinase p85-alpha subunit (PIK3R1)

PIK3R1 ranks first in importance among the identified targets in terms of degree is the second most important in terms of betweenness centrality (Table 2). Class IA PI3Ks consist of two main subunits: a catalytic subunit (p110) and a regulatory subunit (p85 or p85-like). PI3-kinase p85-alpha subunit (PIK3R1) is expressed ubiquitously in all tissues (Jean and Kiger, 2014; Rathinaswamy and Burke, 2020). It stabilizes and inhibits p110 activity and acts as an adaptor for PI3K to interact with IRS and growth factor receptors. Excessive amounts of monomeric PIK3R1 can compete with active dimeric PI3K for binding to IRS, thereby inhibiting insulin signaling. PIK3R1 contains two Src homology 2 (SH2) domains, on the N-terminal (nSH2) and C-terminal (cSH2) regions. The nSH2 domain is mandatory for the inhibition of p110 activity (Tsay and Wang, 2023).

**FIGURE 11:**
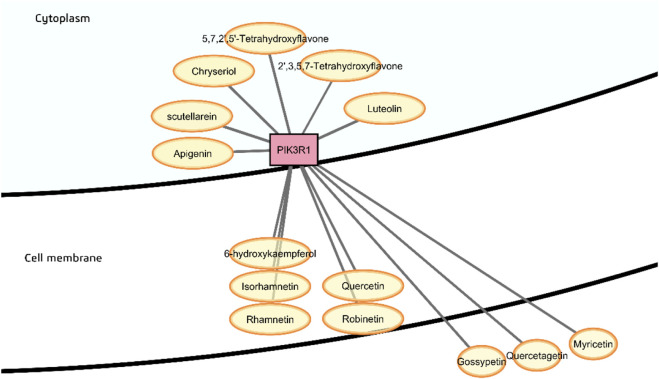
Probable binders of PI3-kinase p85-alpha subunit (PIK3R1).

Phosphoinositides serve as important signaling molecules that directly interact with membrane proteins or recruit cytoplasmic proteins to the membrane. Phosphatidylinositol-4,5-bisphosphate 3-kinase (PI3K) is located just below the inner leaflet of the inner membrane, and phosphorylates phosphatidylinositol-4,5-bisphosphate (PIP2) to phosphatidylinositol-3,4,5-trisphosphate (PIP3). The phytochemical class predicted to be probable binders of PIK3R1, the regulatory subunit of PI3K, are flavonoids (Figure 11), and most have favorable calculated consensus log *P* values that may enable entry across the cell membrane, however, myricetin, gossypetin, and quercetagetin were calculated to have consensus log *P* < 1 as well as low GI absorption and so are less likely to interact with PIK3R1. These three phytochemicals are also predicted to target the insulin receptor, which is at the surface of the plasma membrane (with both extracellular and cytoplasmic domains), and if absorbed after ingestion, are more likely to interact with INSR on the cell surface rather than PIK3R1.

In a previous study, the x-ray crystal structure of the ATP binding pocket of PIK3R1 occupied by a potent and selective inhibitor of the α isoform demonstrated hydrogen bonding and hydrophobic interactions with Val851, Tyr836, Asp810, Ile800, Lys802, Pro778, Met772, Asp933, Gln859 and Ser854 (Furet et al., 2013). In the current study, various flavonoids, namely, apigenin, scutellarein, chryseriol, luteolin, 2′,5,5′,7-tetrahydroxyflavone, 2′,3,5,7-tetrahydroxyflavone, 6-hydroxykaempferol, isorhamnetin, rhamnetin, quercetin and robinetin, as well as ellagic acid, were predicted to have probable binding with PIK3R1 and high gastrointestinal absorption. In particular, in the best docking pose of 2′,5,5′,7-tetrahydroxyflavone, it is seen to possibly interact with PIK3R1 *via* hydrogen bonds with Val851, Tyr836, Asp933, and Ser854 (Supplementary Figure S8). This is consistent with a previous study on the anti-breast cancer effect of Huangqi-Danggui herb pair that identified PIK3R1 as the core target, verified using an *in vitro* and *in vivo* mouse model (Liu et al., 2024). Molecular docking of the flavonoids quercetin, jaranol, isorhamnetin, kaempferol and calycosin with PIK3R1 revealed similar interactions as in the present study.

#### 4.4.2 Insulin receptor (INSR)

**FIGURE 12:**
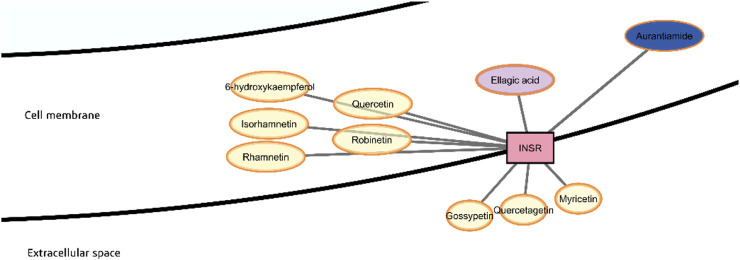
Probable binders of insulin receptor (INSR).

Insulin receptor (INSR) ranks third in importance among the different protein targets in terms of degree, betweenness and closeness centrality (Table 2). The phytochemicals predicted to be strong binders of the insulin receptor are, in general, flavonoids, namely, 6-hydroxykaempferol, isorhamnetin, quercetin, rhamnetin, robinetin, quercetagetin, myricetin, and gossypetin, plus ellagic acid, a phenolic acid (Figure 12). However, quercetagetin, myricetin, and gossypetin are predicted to have low GI absorption.

The insulin receptor is a glycosylated, disulfide-linked heterodimeric α2β2 transmembrane protein expressed in the cell membrane of insulin-sensitive cells. When insulin binds to an extracellular α chain, the two β chains unwind into the intracellular space and activate each other through autophosphorylation at tyrosine protein kinase domains. This allows interaction with the insulin receptor substrate (IRS), which is phosphorylated by INSR at four Tyr residues.

In a previous study, a chaetochromin derivative named 4548-G05 was found experimentally to be a potent insulin receptor agonist that induces phosphorylation of the insulin receptor (Qiang et al., 2014). The compound 4548-G05 is a naphthopyranone, bearing some structural similarity to flavonoids being also polyphenolic. Molecular docking previously suggested that 4548-G05 can bind to the cytoplasmic domain of the insulin receptor where tyrosine kinase activity occurs, demonstrating hydrogen bonds to Asp1177 and Ser1033. For other ligands of the INSR in the cytoplasmic region, hydrogen bonds with other residues such as Met1106, Glu1104, Arg1163, Lys1057 have also been observed in the inhibitor binding pocket (Kumar et al., 2022). All these possible interactions are also seen in the predicted best pose of 6-hydroxykaempferol with INSR. For aurantiamide, there are possible hydrophobic interaction with Asp1177 and hydrogen bonding with the adjacent Gly1176 instead (Supplementary Figure S9).

#### 4.4.3 Protein-tyrosine phosphatase 1B (PTPN1 or PTP1B)

**FIGURE 13:**
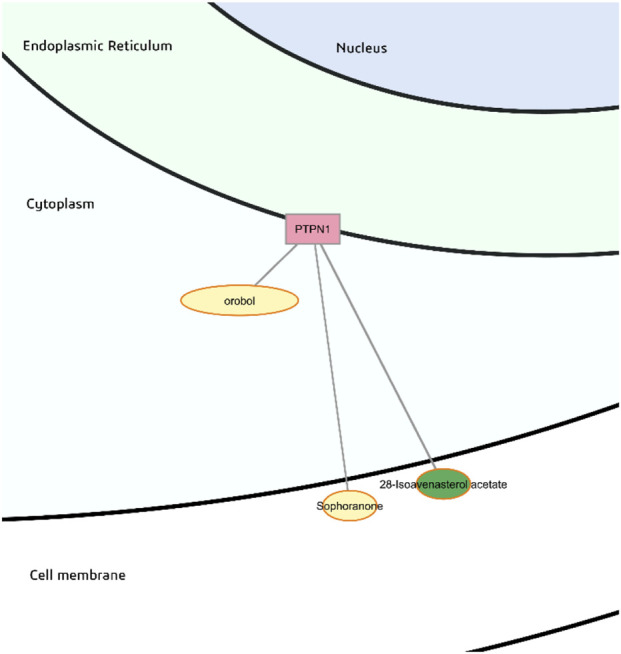
Probable binders of protein-tyrosine phosphatase 1B (PTPN1).

The isoflavonoid orobol with predicted high gastrointestinal absorption and moderate bioavailability is predicted to be a probable binder of PTPN1. Sophoranone and 28-isoavenasterol acetate may also probably bind PTPN1 but these have low predicted gastrointestinal absorption (Figure 13).

While activation of the insulin receptor (INSR) involves phosphorylation, protein-tyrosine phosphatase 1B (PTPN1 or PTP1B) contradicts this through converse phosphatase activity on both INSR and IRS. PTPN1 hydrolyzes tyrosine phosphorylation and is a negative regulator of insulin signaling. It counteracts the action of the insulin receptor as well as other receptor tyrosine kinases (RTKs) such as platelet-derived growth factor (PDGF), epithelial growth factor (EGF), and Janus kinase 2 (JAK2). It has also been shown that PTPN1 inhibition suppresses ER stress-induced apoptosis and cell cycle arrest in endothelial cells (Abdelsalam et al., 2021). Because of the role of these RTKs related to cell growth, differentiation, and metabolism, PTPN1 inhibition has been of interest in diseases such as diabetes, obesity, cardiovascular ischemic conditions, and various types of cancer.

PTPN1 is primarily an ER-bound enzyme with its catalytic domain oriented towards the cytosol and anchored onto the endoplasmic reticulum membrane *via* a hydrophobic region in its C-terminal tail. Its location may seem curious as it can interact with a variety of membrane-bound substrates, but it has been found that its location supports its role in endocytosis of RTKs (Stuible and Tremblay, 2010). PTPN1 interacts with its substrates at the tip of dynamic ER tubules that extend to the plasma membrane/substrate interface (Monteleone et al., 2012), and also within endosomal compartments to assist in trafficking of the receptor to the plasma membrane in the context of recycling (Romsicki et al., 2004) or for lysosomal degradation when downregulating RTKs.

Relevant residues that are necessary for the function of PTPN1 include the catalytic residue Cys215 located on its PTP loop. Once the substrate is bound to Arg221, PTPN1 undergoes a conformational change allowing Asp181 on its WPD loop to move closer to the active site and perform a proton transfer to facilitate the reaction (Choy et al., 2017). As can be seen in Supplementary Figure S10, the best docking pose of orobol shows possible hydrogen bonding interactions with the three key residues Cys215, Asp181 and Arg221.

### 4.5 Ubiquity of phytochemicals

Ubiquity of phytochemicals is worth considering to predict a plant’s distinct effect on the disease of interest. The COlleCtion of Open NatUral producTs (COCONUT) database contains over 400,000 non-redundant natural products, and considered to be the most complete and up-to-date continually curated database for natural products (de Azevedo et al., 2024; Sorokina et al., 2021). In this study, phytochemicals with number of occurrences in various plants greater than or equal to that of quinic acid, one of the phytochemicals present in *M. oleifera* and a precursor in the shikimic acid pathway that occurs in all plants, were considered as ubiquitous (Supplementary Table S11). The shikimic acid pathway is necessarily present in all plants as it is the basis for the biosynthesis of aromatic amino acids. It has been called a “gateway of metabolic diversity” because fungi, algae, and plants exploit it in their specialized metabolism (Shende et al., 2024).

**FIGURE 14:**
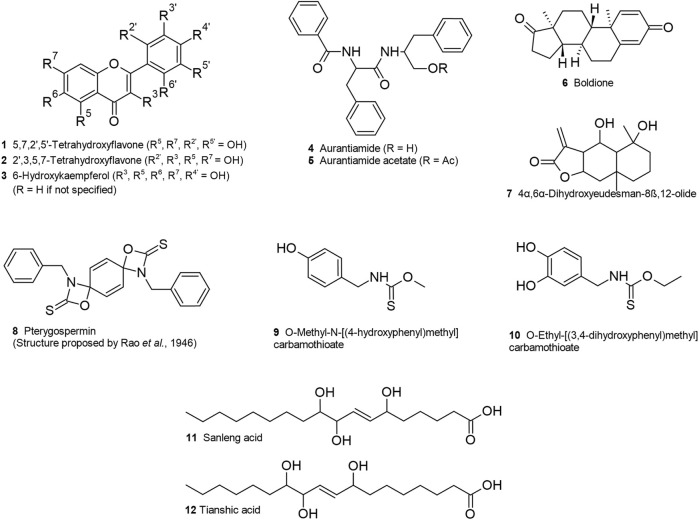
Chemical structures of non-ubiquitous *Moringa oleifera* phytochemicals with predicted high gastrointestinal absorption, moderate bioavailability, and probable binders to insulin resistance targets.

The list of non-ubiquitous *M. oleifera* phytochemicals that are probable binders to insulin resistance targets and, with predicted high GI absorption and moderate bioavailability, are listed in Table 4, and their chemical structures are illustrated in Figure 14.

**TABLE 4:** Non-ubiquitous *Moringa oleifera* phytochemicals with high gastrointestinal absorption that are probable binders to insulin resistance targets.

Phytochemical	Class	Method of structure determination	Predicted target	Predicted affinity (kcal/mol)	Previous reported activity	References
Boldione	Steroid	UHPLC-MS	HSD11B1	−11.3	Anabolic androgenic steroid	Tarkowská (2019), Hong et al. (2023)
Aurantiamide acetate	Peptide derivative	2D NMR and MS	HSD11B1	−10.2	Antitumor, anti-inflammatory, anti-neuro-inflammatory, antiviral, analgesic, suppresses TNF and IL-2, cathepsin inhibitor	Isshiki et al. (2001), Sashidhara et al. (2009), Yoon et al. (2014), Zhou et al. (2017), Fang et al. (2022), Yang et al. (2023)
Aurantiamide	Peptide derivative	Inference (deacetylation product of aurantiamide acetate)	HSD11B1 INSR	−9.7 −9.3	Anti-inflammatory, renal necroptosis suppression, GRPR antagonist, suppresses activation of NLRP3 inflammasome in Alzheimer’s disease	Shen et al. (2023), He et al. (2024)
Pterygospermin	Thiocarbamate	Inference	HSD11B1	−10.0	Antibacterial through inhibition of transaminase	Rao et al. (1946), Kurup et al. (1954)
O-Ethyl [(3,4-dihydroxyphenyl) methyl] carbamothioate	Thiocarbamate	Inference (aglycone of O-ethyl-4-[(a-L-rhamno pyranosyloxy)-3-hydroxy benzyl] thiocarbamate)	NOS3	−7.2	Hypotensive (for glycoside)	Faizi et al. (1998)
O-Methyl N-[(4-hydroxyphenyl) methyl] carbamothioate	Thiocarbamate	Inference (aglycone of niazinin)	NOS3	−6.9	Hypotensive (for glycoside)	Faizi et al. (1992)
4α,6α-Dihydroxy-eudesman-8β,12-olide	Sesquiterpenoid	UPLC-QTOF-MSe	HSD11B1	−8.2	Cytotoxic against human tumor cell lines	Park and Kim (2007), Lin et al. (2019)
Sanleng acid	Fatty acid derivative	UPLC-QTOF-MSe	TNF-α	−8.1	COX-2 suppression	Lin et al. (2019), Zhu et al. (2024)
Tianshic acid	Fatty acid derivative	UPLC-QTOF-MSe	TNF-α	−8.1	Antifungal, Induction of alkaline phosphatase activity of osteoblast cells	Lin et al. (2019), Sang et al. (2002), Yang et al. (2006)
5,7,2′5′-Tetrahydroxy-flavone	Flavonoid (flavone)	UPLC-QTOF-MSe	PIK3R1	−8.6	Inhibition of adipocyte differentiation and NO production in macrophage cells	Yang et al. (2011), Lin et al. (2019)
2′,3,5,7-Tetrahydroxy-flavone	Flavonoid (flavone)	UHPLC-MS	PIK3R1	−8.5	Phosphoinositide-dependent kinase 1 inhibition; activity against antibiotic-resistant bacteria; Inhibition of amyloid β42 aggregation	Xu and Lee (2001), Hanaki et al. (2016), Kang et al. (2017), Hong et al. (2023)
6-Hydroxy-kaempferol	Flavonoid (flavonol)	UPLC-QTOF-MSe	INSR PIK3R1	−8.4 −8.4	Tendon fibroblast proliferator; Tyrosinase, α-glucosidase inhibitor	Gao et al. (2007), Lin et al. (2019), Gutiérrez-González et al. (2021), Mok et al. (2022)

Many flavonoids are ubiquitous and found in a wide array of plant-based foods including fruits, vegetables, tea, and wine. It is well known that a diet rich in flavonoids is associated with lower insulin resistance or lower incidence of type 2 diabetes (Jacques et al., 2013; Jennings et al., 2014; Bondonno et al., 2021). This effect was typically attributed to the antioxidant activity of flavonoids and ability to scavenge free radicals. It is now known that flavonoids can also inhibit inflammatory signaling and activate the translocation of glucose transporters (Al-Ishaq et al., 2019; Ren et al., 2019). In the context of this study, numerous flavonoids are predicted to have probable binding with multiple targets related to insulin resistance, namely, INSR, PIK3R1, and PTPN1, all proximal to the function and regulation of the metabolic PI3K pathway, as well as PPARα. The influence of various ubiquitous flavonoids on these multiple targets, acting synergistically in a multi-component, multi-target manner, may be the reason for the association between flavonoid-rich diets in general, regardless of plant source, and lower insulin resistance. The flavonoids 2′,5,5′,7-tetrahydroxyflavone (**1**), 2′,3,5,7-tetrahydroxyflavone (**2**), and 6-hydroxykaempferol (**3**) were listed in 2, 4, and 11 species, respectively, in the COCONUT database. Less ubiquitous than the other flavonoids, they were similarly predicted to target the metabolic PI3K cluster.

Apart from the flavonoids listed in Table 4, the other non-ubiquitous phytochemicals have probable targets involved in the regulation of lipid storage, with the exception of aurantiamide (**4**) which is also predicted to target the insulin receptor.

Aurantiamide acetate (**5**) and aurantiamide are peptide derivatives, with 15 and 3 occurrences in the COCONUT database, respectively. Aurantiamide acetate has been recognized to possess analgesic activity in mice, although 1,3-dibenzylurea, another phytochemical included in this study but predicted to be a probable nonbinder, seemed to be more potent (Sashidhara et al., 2009). In this study, both aurantiamide acetate and aurantiamide were predicted to target HSD11B1.

Boldione (**6**) is an anabolic steroid that is also used in the veterinary care of horses to enhance muscle growth and appetite. Although considered an animal steroid hormone, it has been found to be natural constituents of *Pinus halepensis* and *Tribulus terrestris* (Tarkowská, 2019). In another study, boldione was predicted to be one of the main phytochemicals of *M. oleifera* that exert an antidiabetic effect using network pharmacology, although HSD11B1 was not identified as one of the main targets (Hong et al., 2023).

The sesquiterpenoid lactone 4α,6α-dihydroxyeudesman-8β,12-olide (**7**) has been identified in 2 species as listed in the COCONUT database. This compound has been reported to possess cytotoxic activity against human tumor cell lines (Park and Kim, 2007).

Glucosinolates are a known component of many species under the order Brassicales, including *M. oleifera*. As glucosinolates are rapidly converted to isothiocyanates by the enzyme myrosinase, it is not likely that glucosinolates are the actual active principles that account for the pharmacological effects of *M. oleifera*. In this study, none of the glucosinolates was shown to be probably active for insulin resistance.

Pterygospermin (**8**) is a thiocarbamate that has been characterized only in *M. oleifera*, and while it may also be present in other Moringa species, research is limited only to a few species (Abd Rani et al., 2018). It was proposed to be the antibiotic principle in this plant (Rao et al., 1946; Kurup et al., 1954). The proposed chemical structure of pterygospermin, however, has been disputed to be not stable enough to exist at ambient conditions, and the antibacterial activity of the plant has been attributed to isothiocyanates instead (Horwath and Benin, 2011).

Isothiocyanates such as moringin have previously been shown to possess various pharmacological activities, and isothiocyanate-rich *M. oleifera* extracts have been reported to improve insulin resistance in mice models (Waterman et al., 2015). Carbamates and thiocarbamate glycosides of *M. oleifera* have also been demonstrated to possess hypotensive activity (Faizi et al., 1994). In this study, the thiocarbamate aglycones, O-methyl N-[(4-hydroxyphenyl)methyl] carbamothioate (**9**) and O-ethyl [(3,4-dihydroxyphenyl)methyl] carbamothioate (**10**), were predicted to be probable binders of eNOS. As already discussed in Section 4.3.5, we have proposed a link between the blood-pressure lowering effect of *M. oleifera* leaf extract and eNOS, and that aglycones of thiocarbamate glycosides of *M. oleifera* may be the actual bioactive forms of the compounds that specifically target it, rather than isothiocyanates or thiocarbamate glycosides. In the context of insulin resistance, eNOS activity is relevant because NO acts as an anti-inflammatory mediator.

Interestingly, thiazolidinediones (TZDs), a class of drugs used for treatment of insulin resistance, share structural similarity with thiourea and thiocarbamates, and there are synthetic routes for TZDs that utilize these as starting materials (Chadha and Silakari, 2018; Basak et al., 2024). The pharmacological target of TZDs is PPARγ.

Sanleng acid (**11**) and tianshic acid (**12**) are listed under 1 and 3 species, respectively, in the COCONUT database. They are fatty acid derivatives and are predicted in this study to have probable binding with TNF-α, implying that they may have anti-inflammatory effects. Influence of different types of fatty acids on immune response have been the subject of enquiry over the years, and it is a general opinion that monounsaturated and polyunsaturated fatty acids (particularly n-3) have anti-inflammatory effects. Information on the effect of fatty acid derivatives is still emerging. Sanleng acid or (*E*)-6,9,10-trihydroxyoctadec-7-enoic acid was found to have COX-2 inhibitory effect (Zhu et al., 2024). Tianshic acid or (*E*)-8,11,12-trihydroxyoctadec-9-enoic acid was identified as one of the components of *Allium tuberosum* extract, which was shown to have preventive and anti-inflammatory effect in TNF-α-stimulated human umbilical vein endothelial cells (Hur and Lee, 2017). Both sanleng acid and tianshic acid are trihydroxy monounsaturated fatty acids. Specialized pro-resolving mediators (SPMs), so-called for their role in resolution of inflammation, are also hydroxy derivatives of the essential fatty acids EPA, DHA or AA from the action of lipoxygenases (LOX) or cyclooxygenases (COX), then subsequent reduction or epoxide hydrolysis (Maliha et al., 2024). This structural resemblance to SPMs may be relevant to the supposed anti-inflammatory effects of sanleng and tianshic acids.

All the non-ubiquitous phytochemicals with high GI absorption and determined by this study to be probable binders of insulin resistance targets have already been reported to possess various protective activities, but not anti-insulin resistance or antidiabetic activity (except for boldione). The interactions highlighted in Table 4 may distinguish the effect of *M. oleifera* against insulin resistance *versus* that of other plants and are worth looking into for further *in vivo*, *ex vivo*, or *in vitro* validation.

### 4.6 Recommendations for validation studies

Feasibility of target-based assays for the validation of the predicted effects herein described are subject to the availability of standards in their pure form. To this end, commercial availability of the aforementioned moringa phytochemicals based on listing of at least three chemical vendors in PubChem are listed in Supplementary Table S12, along with synthetic accessibility estimated by SwissADME.

Being of interest in various diseases, the aforementioned targets have already been investigated using various assays, examples of which are listed in Supplementary Table S13. Most of these are *in vitro* assays, but it is intriguing to note the use of an *ex vivo* immuno-radiometric scintillation proximity assay for HSD11B1 where a compound was tested directly on biopsied human adipose tissue (Zhang et al., 2022). This is particularly applicable in the context of insulin resistance where adiposity is a major contributor and provides a way to test on human subjects without the risk of exposure. HSD11B1 was predicted to be the most important target in terms of betweenness and second most important in terms of degree. It is more likely to be the subject of the unique effect of *M. oleifera* because of the variety of phytochemical classes that may target it, including the non-ubiquitous compounds boldione, aurantiamide acetate, and 4α,6α-dihydroxyeudesman-8β,12-olide that are commercially available in their pure form for testing.

It has been mentioned that aglycones of thiocarbamate glycosides of *M. oleifera* may be the actual bioactive forms of the compounds. Thiocarbamates have been demonstrated in other studies to exhibit hypotensive effects (Faizi et al., 1992; Faizi et al., 1994, Faizi et al., 1998) and it would be well worthwhile to pinpoint the actual target, likely eNOS based on the results of this *in silico* study. The thiocarbamates O-ethyl [(3,4-dihydroxyphenyl)methyl] carbamothioate and O-methyl N-[(4-hydroxyphenyl)methyl] carbamothioate are not commercially available, but their synthesis may be feasible based on computed synthetic accessibility of 2.03 and 1.55, respectively. Practical evaluation of synthetic accessibility may differ, however, among medicinal chemists vis-à-vis the estimated value from SwissADME.

Aside from non-ubiquitous phytochemicals, we should also consider the other flavonoids which are readily available and cheaper. The notion of ubiquity was introduced as a way to reduce the number of candidate compounds and differentiate the unique effect of the plant from other plants. These flavonoids were not prioritized because they are ubiquitous, following the definition based on comparison with quinic acid. However, this does not mean that these compounds do not contribute to bioactivity. Studying the flavonoid group can be very instructive since we can delve into the phenomena of synergistic effects where the combined effect is greater than singular effects.

The top-ranked phytochemicals may be used as chemical markers for the plant in future work. This can be done with a small amount of pure standards using NMR, HPTLC and LC-MS/MS.

## 5 Conclusion

*Moringa oleifera* phytochemicals were classified as probable binders or probable nonbinders of targets relevant to insulin resistance using a modified network pharmacology and molecular docking approach where optimal thresholds for binding affinities from docking experiments were calculated using receiver operating characteristic (ROC) curves. Considering only probable binders of insulin resistance targets, there were 46 interactions between 35 phytochemicals with 8 hub targets in 2 clusters, namely a) HSD11B1, TNF-α, PPARα, PPARγ, and eNOS in the cluster for regulation of lipid storage, and b) PIK3R1, INSR, and PTPN1 in the cluster involving the metabolic PI3K pathway leading to glucose uptake. Ubiquity of phytochemicals was estimated based on the number of species where it is present in the COCONUT database in comparison with quinic acid. Flavonoids, many of which are ubiquitous in plants, are predicted to mainly influence the targets in the cluster involving the metabolic PI3K pathway, as well as PPARα, which may explain the positive effects of a flavonoid-rich diet against insulin resistance. Non-ubiquitous *M. oleifera* phytochemicals with predicted high gastrointestinal absorption, moderate bioavailability and probable binding to relevant insulin resistance targets include 2′,5,5′,7-tetrahydroxyflavone, 2′,3,5,7-tetrahydroxyflavone, and 6-hydroxykaempferol (flavonoids); aurantiamide and aurantiamide acetate (peptide derivatives); boldione (a steroid); 4α,6α-dihydroxyeudesman-8β,12-olide (a sesquiterpenoid lactone); pterygospermin, O-methyl-N-[(4-hydroxyphenyl)methyl]carbamothioate, and O-ethyl-[(3,4-dihydroxyphenyl)methyl]carbamothioate, (thiocarbamates); and sanleng acid and tianshic acid (fatty acid derivatives). Aglycones of thiocarbamate glycosides of *M. oleifera* may be the actual bioactive forms of sulfur-containing compounds that ameliorate insulin resistance by targeting eNOS. The aforementioned may be considered as candidates for key bioactives that may be used as chemical markers of *M. oleifera*. The methods outlined in this paper are offered as a further refinement of the network pharmacology approach on the way to its standardization and wider acceptance. *In vitro*, *in vivo*, or *ex vivo* validation is recommended to verify these findings.

## Data Availability

The original contributions presented in the study are included in the article/Supplementary Material, further inquiries can be directed to the corresponding author.

## References

[B1] Abd Rani N. Z. Husain K. Kumolosasi E. (2018). *Moringa* genus: a review of phytochemistry and pharmacology. Front. Pharmacol. 9, 108. 10.3389/fphar.2018.00108 29503616 PMC5820334

[B2] Abdelsalam S. S. Pasha M. El-Gamal H. Hasan M. Elrayess M. A. Zeidan A. (2021). Protein tyrosine phosphatase 1B inhibition improves endoplasmic reticulum stress-impaired endothelial cell angiogenic response: a critical role for cell survival. Mol. Med. Rep. 24, 665. 10.3892/mmr.2021.12304 34296297

[B3] Abulizi A. Camporez J.-P. Zhang D. Samuel V. T. Shulman G. I. Vatner D. F. (2019). Ectopic lipid deposition mediates insulin resistance in adipose specific 11β-hydroxysteroid dehydrogenase type 1 transgenic mice. Metabolism 93, 1–9. 10.1016/j.metabol.2018.12.003 30576689 PMC6401251

[B4] Advanced Chemistry Development, Inc. (ACD/Labs) (2021). ACD/ChemSketch. Toronto, Ontario, Canada.

[B5] Aekthammarat D. Pannangpetch P. Tangsucharit P. (2019). *Moringa oleifera* leaf extract lowers high blood pressure by alleviating vascular dysfunction and decreasing oxidative stress in L-NAME hypertensive rats. Phytomedicine 54, 9–16. 10.1016/j.phymed.2018.10.023 30668387

[B6] Agarwal R. Smith J. C. (2023). Speed vs accuracy: effect on ligand pose accuracy of varying box size and exhaustiveness in AutoDock vina. Mol. Inf. 42, 2200188. 10.1002/minf.202200188 36262028

[B7] Al-Ishaq R. K. Abotaleb M. Kubatka P. Kajo K. Büsselberg D. (2019). Flavonoids and their anti-diabetic effects: cellular mechanisms and effects to improve blood sugar levels. Biomolecules 9, 430. 10.3390/biom9090430 31480505 PMC6769509

[B8] Alberts B. Johnson A. Lewis J. Raff M. Roberts K. Walter P. (2002). “The transport of molecules between the nucleus and the cytosol,” in Molecular biology of the cell (New York, NY: Garland Science).

[B9] Alzamil H. (2020). Elevated serum TNF-α is related to obesity in type 2 diabetes mellitus and is associated with glycemic control and insulin resistance. J. Obes. 2020, 5076858. 10.1155/2020/5076858 32089876 PMC7013317

[B10] Anwar F. Latif S. Ashraf M. Gilani A. H. (2007). *Moringa oleifera*: a food plant with multiple medicinal uses. Phytother. Res. 21, 17–25. 10.1002/ptr.2023 17089328

[B11] Artis D. R. Lin J. J. Zhang C. Wang W. Mehra U. Perreault M. (2009). Scaffold-based discovery of indeglitazar, a PPAR pan-active anti-diabetic agent. Proc. Natl. Acad. Sci. U. S. A. 106, 262–267. 10.1073/pnas.0811325106 19116277 PMC2629228

[B12] AutoDock Vina 1.12 Manual (2011). AutoDock Vina 1.12 Manual WWW document.Available online at: https://vina.scripps.edu/manual/(Accessed July 17, 25).

[B13] Autodock Vina 1.2.0 Documentation (2021). Autodock Vina 1.2.0 Documentation WWW document. Available online at: https://autodock-vina.readthedocs.io/en/latest/vina.html (Accessed July 17, 25).

[B14] Bao Y. Xiao J. Weng Z. Lu X. Shen X. Wang F. (2020). A phenolic glycoside from *Moringa oleifera* Lam. improves the carbohydrate and lipid metabolisms through AMPK in db/db mice. Food Chem. 311, 125948. 10.1016/j.foodchem.2019.125948 31877545

[B15] Basak S. Murmu A. Matore B. W. Roy P. P. Singh J. (2024). Thiazolidinedione an auspicious scaffold as PPAR-γ agonist: its possible mechanism to manoeuvre against insulin resistant diabetes mellitus. Eur. J. Med. Chem. Rep. 11, 100160. 10.1016/j.ejmcr.2024.100160

[B16] Berman H. M. Westbrook J. Feng Z. Gilliland G. Bhat T. N. Weissig H. (2000). The protein data bank. Nucleic Acids Res. 28, 235–242. 10.1093/nar/28.1.235 10592235 PMC102472

[B18] Bodhini D. Mohan V. (2018). Mediators of insulin resistance and cardiometabolic risk: newer insights. Indian J. Med. Res. 148, 127–129. 10.4103/ijmr.IJMR_969_18 30381534 PMC6206760

[B19] Bondonno N. P. Dalgaard F. Murray K. Davey R. J. Bondonno C. P. Cassidy A. (2021). Higher habitual flavonoid intakes are associated with a lower incidence of diabetes. J. Nutr. 151, 3533–3542. 10.1093/jn/nxab269 34313759 PMC8562076

[B22] Briones Y. L. Young A. T. Dayrit F. M. De Jesus A. J. Rojas N. R. L. (2021). Visualizing phytochemical-protein interaction networks: momordica charantia and cancer. Front. Bioinform 1, 768886. 10.3389/fbinf.2021.768886 36303742 PMC9580883

[B23] Chadha N. Silakari O. (2018). “Chapter 5 - Thiazolidine-2,4-Dione: a potential weapon for targeting multiple pathological conditions,” in Key heterocycle cores for designing multitargeting molecules. Editor Silakari O. (Amsterdam, Netherlands: Elsevier), 175–209. 10.1016/B978-0-08-102083-8.00005-4

[B24] Chang P. W. Newman T. B. (2024). Receiver operating characteristic (ROC) curves: the basics and beyond. Hosp. Pediatr. 14, e330–e334. 10.1542/hpeds.2023-007462 38932727

[B25] Chapman K. Holmes M. Seckl J. (2013). 11β-Hydroxysteroid dehydrogenases: intracellular gate-keepers of tissue glucocorticoid action. Physiol. Rev. 93, 1139–1206. 10.1152/physrev.00020.2012 23899562 PMC3962546

[B26] Che X. Liu Q. Zhang L. (2023). An accurate and universal protein-small molecule batch docking solution using Autodock Vina. Results Eng. 19, 101335. 10.1016/j.rineng.2023.101335

[B27] Chédotal H. Narayanan D. Povlsen K. Gotfredsen C. H. Brambilla R. Gajhede M. (2023). Small-molecule modulators of tumor necrosis factor signaling. Drug Discov. Today 28, 103575. 10.1016/j.drudis.2023.103575 37003513

[B28] Chen S.-L. Yu H. Luo H.-M. Wu Q. Li C.-F. Steinmetz A. (2016). Conservation and sustainable use of medicinal plants: problems, progress, and prospects. Chin. Med. 11, 37. 10.1186/s13020-016-0108-7 27478496 PMC4967523

[B29] Choy M. S. Li Y. Machado L. E. S. F. Kunze M. B. A. Connors C. R. Wei X. (2017). Conformational rigidity and protein dynamics at distinct timescales regulate PTP1B activity and allostery. Mol. Cell 65, 644–658.e5. 10.1016/j.molcel.2017.01.014 28212750 PMC5325675

[B31] Daina A. Zoete V. (2016). A BOILED‐Egg to predict gastrointestinal absorption and brain penetration of small molecules. ChemMedChem 11, 1117–1121. 10.1002/cmdc.201600182 27218427 PMC5089604

[B32] Daina A. Michielin O. Zoete V. (2017). SwissADME: a free web tool to evaluate pharmacokinetics, drug-likeness and medicinal chemistry friendliness of small molecules. Sci. Rep. 7, 1–13. 10.1038/srep42717 28256516 PMC5335600

[B33] Daina A. Michielin O. Zoete V. (2019). SwissTargetPrediction: updated data and new features for efficient prediction of protein targets of small molecules. Nucleic Acids Res. 47, W357–W364. 10.1093/nar/gkz382 31106366 PMC6602486

[B34] Dayal B. Yannamreddy V. R. Amin R. Lea M. A. Attygalle A. B. (2013). Bioactive compounds in *Moringa oleifera*: isolation, structure elucidation, and their antiproliferative properties. ACS Symp. Ser. Am. Chem. Soc., Trop. Subtropical Fruits Flavors, Color, Health Benefits 1129, 203–219. 10.1021/bk-2013-1129.ch013

[B35] de Azevedo D. Q. Campioni B. M. Pedroz Lima F. A. Medina-Franco J. L. Castilho R. O. Maltarollo V. G. (2024). A critical assessment of bioactive compounds databases. Future Med. Chem. 16, 1029–1051. 10.1080/17568919.2024.2342203 38910575 PMC11221550

[B36] Dedio J. König P. Wohlfart P. Schroeder C. Kummer W. Müller-Esterl W. (2001). NOSIP, a novel modulator of endothelial nitric oxide synthase activity. FASEB J. 15, 79–89. 10.1096/fj.00-0078com 11149895

[B37] Deng W. Verlinde C. L. M. J. (2008). Evaluation of different virtual screening programs for docking in a charged binding pocket. J. Chem. Inf. Model. 48, 2010–2020. 10.1021/ci800154w 18821750 PMC2761750

[B39] Dujic T. Bego T. Mlinar B. Semiz S. Malenica M. Prnjavorac B. (2012). Association between 11β-hydroxysteroid dehydrogenase type 1 gene polymorphisms and metabolic syndrome in Bosnian population. Biochem. Med. 22, 76–85. 10.11613/BM.2012.008 22384521 PMC4062331

[B40] Duranti G. Maldini M. Crognale D. Sabatini S. Corana F. Horner K. (2021). *Moringa oleifera* leaf extract influences oxidative metabolism in C2C12 myotubes through SIRT1-PPARα pathway. Phytomed. Plus 1, 100014. 10.1016/j.phyplu.2020.100014

[B41] Eberhardt J. Santos-Martins D. Tillack A. F. Forli S. (2021). AutoDock Vina 1.2.0: new docking methods, expanded force field, and Python bindings. J. Chem. Inf. Model. 61, 3891–3898. 10.1021/acs.jcim.1c00203 34278794 PMC10683950

[B42] Eilert U. Wolters B. Nahrstedt A. (1981). The antibiotic principle of seeds of *Moringa oleifera* and *Moringa stenopetala* . Planta Med. 42, 55–61. 10.1055/s-2007-971546 7255568

[B44] Fahed M. Jaoudeh M. G. A. Merhi S. Mosleh J. M. B. Ghadieh R. Hayek S. A. (2020). Evaluation of risk factors for insulin resistance: a cross sectional study among employees at a private university in Lebanon. BMC Endocr. Disord. 20, 1–14. 10.1186/s12902-020-00558-9 32522257 PMC7288486

[B45] Fahey J. W. Wade K. L. Stephenson K. K. Shi Y. Liu H. Panjwani A. A. (2019). A strategy to deliver precise oral doses of the glucosinolates or isothiocyanates from *Moringa oleifera* leaves for use in clinical studies. Nutrients 11, 1547. 10.3390/nu11071547 31323988 PMC6682957

[B46] Faizi S. Siddiqui B. Saleem R. Siddiqui S. Aftab K. Gilani A. (1992). Isolation and structure elucidation of novel hypotensive agents, Niazinin A, Niazinin B, Niazimicin and Niaziminin A + B from *Moringa oleifera*: the first naturally occurring thiocarbamates. J. Chem. Soc. Perkin Trans. 1, 3237–3241 1. 10.1039/p19920003237

[B47] Faizi S. Siddiqui B. S. Saleem R. Siddiqui S. Aftab K. Gilani A. H. (1994). Isolation and structure elucidation of new nitrile and mustard oil glycosides from *Moringa oleifera* and their effect on blood pressure. J. Nat. Prod. 57, 1256–1261. 10.1021/np50111a011 7798960

[B48] Faizi S. Siddiqui B. S. Saleem R. Aftab K. Shaheen F. Gilani A.-H. (1998). Hypotensive constituents from the pods of *Moringa oleifera* . Planta Med. 64, 225–228. 10.1055/s-2006-957414 9581519

[B49] Fang Z. Fang J. Gao C. Wu Y. Yu W. (2022). Aurantiamide acetate ameliorates lung inflammation in lipopolysaccharide-induced acute lung injury in mice. Biomed. Res. Int. 2022, 3510423. 10.1155/2022/3510423 36046440 PMC9424011

[B50] Farooq F. Rai M. Tiwari A. Khan A. Farooq S. (2012). Medicinal properties of *Moringa oleifera*: an overview of promising healer. J. Med. Plants Res. 6, 4368–4374. 10.5897/JMPR12.279

[B51] Favia A. D. Masetti M. Recanatini M. Cavalli A. (2011). Substrate binding process and mechanistic functioning of type 1 11β-Hydroxysteroid dehydrogenase from enhanced sampling methods. PLoS One 6, e25375. 10.1371/journal.pone.0025375 21966510 PMC3179505

[B52] Feinstein W. P. Brylinski M. (2015). Calculating an optimal box size for ligand docking and virtual screening against experimental and predicted binding pockets. J. Cheminform. 7, 18. 10.1186/s13321-015-0067-5 26082804 PMC4468813

[B53] Feng J. Wang X. Ye X. Ares I. Lopez-Torres B. Martínez M. (2022). Mitochondria as an important target of metformin: the mechanism of action, toxic and side effects, and new therapeutic applications. Pharmacol. Res. 177, 106114. 10.1016/j.phrs.2022.106114 35124206

[B54] Ferré P. (2004). The biology of peroxisome proliferator-activated receptors: relationship with lipid metabolism and insulin sensitivity. Diabetes 53, S43–S50. 10.2337/diabetes.53.2007.S43 14749265

[B55] Foti P. Erba D. Spadafranca A. Ciappellano S. Bresciani J. Testolin G. (2006). Daidzein is absorbed by passive transport in isolated small intestine of rats. Nutr. Res. 26, 284–288. 10.1016/j.nutres.2006.06.005

[B56] Frandsen H. B. Jensen S. K. Kristensen N. B. Maribo H. Sørensen J. C. Sørensen H. (2011). “Bioavailability of glucosinolates in feed; uptake and metabolism of intact glucosinolates by pigs,” in Presented at the 13th international Rapeseed Congress, Global Council for Innovation in rapeseed and Canola. (Prague, Czech Republic: Czech Republic).

[B58] Furet P. Guagnano V. Fairhurst R. A. Imbach-Weese P. Bruce I. Knapp M. (2013). Discovery of NVP-BYL719 a potent and selective phosphatidylinositol-3 kinase alpha inhibitor selected for clinical evaluation. Bioorg. Med. Chem. Lett. 23, 3741–3748. 10.1016/j.bmcl.2013.05.007 23726034

[B59] Galuppo M. Giacoppoa S. De Nicola G. R. Iori R. Navarra M. Lombardo G. E. (2014). Antiinflammatory activity of glucomoringin isothiocyanate in a mouse model of experimental autoimmune encephalomyelitis. Fitoterapia 95, 160–174. 10.1016/j.fitote.2014.03.018 24685508

[B60] Gao H. Nishida J. Saito S. Kawabata J. (2007). Inhibitory effects of 5,6,7-Trihydroxyflavones on tyrosinase. Molecules 12, 86–97. 10.3390/12010086 17693955 PMC6149327

[B61] Gao W. Yang G. Liu X. Hu K. Pan J. Wang X. (2025). Network pharmacology and experimental verification to investigate the mechanism of isoliquiritigenin for the treatment of Alzheimer’s disease. Sci. Rep. 15, 4379. 10.1038/s41598-025-88542-y 39910202 PMC11799321

[B62] Gelin M. Delfosse V. Allemand F. Hoh F. Sallaz-Damaz Y. Pirocchi M. (2015). Combining ‘dry’ co-crystallization and *in situ* diffraction to facilitate ligand screening by X-ray crystallography. Acta Cryst. D. 71, 1777–1787. 10.1107/S1399004715010342 26249358

[B63] Gilson M. K. Liu T. Baitaluk M. Nicola G. Hwang L. Chong J. (2016). BindingDB in 2015: a public database for medicinal chemistry, computational chemistry and systems pharmacology. Nucleic Acids Res. 44, D1045–D1053. 10.1093/nar/gkv1072 26481362 PMC4702793

[B64] Gómez-Martínez S. Díaz-Prieto L. E. Vicente Castro I. Jurado C. Iturmendi N. Martín-Ridaura M. C. (2022). *Moringa oleifera* leaf supplementation as a glycemic control strategy in subjects with prediabetes. Nutrients 14, 57. 10.3390/nu14010057 35010932 PMC8746299

[B65] Gomez-Sanchez E. P. Gomez-Sanchez C. E. (2021). 11β-hydroxysteroid dehydrogenases: a growing multi-tasking family. Mol. Cell. Endocrinol. 526, 111210. 10.1016/j.mce.2021.111210 33607268 PMC8108011

[B66] Govindarajulu M. Pinky P. D. Bloemer J. Ghanei N. Suppiramaniam V. Amin R. (2018). Signaling mechanisms of selective PPARγ modulators in Alzheimer’s disease. PPAR Res. 2018, 2010675. 10.1155/2018/2010675 30420872 PMC6215547

[B67] Guevara A. P. Vargas C. Sakurai H. Fujiwara Y. Hashimoto K. Maoka T. (1999). An antitumor promoter from *Moringa oleifera* Lam. Mutat. Res. - Genet. Toxicol. Environ. Mutagen. 440, 181–188. 10.1016/S1383-5718(99)00025-X 10209341

[B68] Gutiérrez-González J. A. Pérez-Vásquez A. Torres-Colín R. Rangel-Grimaldo M. Rebollar-Ramos D. Mata R. (2021). α-Glucosidase inhibitors from *Ageratina grandifolia* . J. Nat. Prod. 84, 1573–1578. 10.1021/acs.jnatprod.1c00105 33857371

[B69] Habtemariam S. (2017). “The African and *Arabian moringa* species: chemistry,” in Bioactivity and therapeutic applications. Oxford, United Kingdom: Elsevier.

[B70] Hanaki M. Murakami K. Akagi K. Irie K. (2016). Structural insights into mechanisms for inhibiting amyloid β42 aggregation by non-catechol-type flavonoids. Bioorg. Med. Chem. 24, 304–313. 10.1016/j.bmc.2015.12.021 26719209

[B71] He R.-B. Li W. Yao R. Xu M.-Y. Dong W. Chen Y. (2024). Aurantiamide mitigates acute kidney injury by suppressing renal necroptosis and inflammation *via* GRPR-dependent mechanism. Int. Immunopharmacol. 139, 112745. 10.1016/j.intimp.2024.112745 39059099

[B73] Hong Z. Xie J. Hu H. Bai Y. Hu X. Li T. (2023). Hypoglycemic effect of *Moringa oleifera* leaf extract and its mechanism prediction based on network pharmacology. J. Future Foods 3, 383–391. 10.1016/j.jfutfo.2023.03.009

[B74] Hopkins A. L. (2007). Network pharmacology. Nat. Biotechnol. 25, 1110–1111. 10.1038/nbt1007-1110 17921993

[B75] Horwath M. Benin V. (2011). Theoretical investigation of a reported antibiotic from the “Miracle Tree” *Moringa oleifera* . Comput. Theor. Chem. 965, 196–201. 10.1016/j.comptc.2011.01.045

[B76] Hsin K.-Y. Matsuoka Y. Asai Y. Kamiyoshi K. Watanabe T. Kawaoka Y. (2016). systemsDock: a web server for network pharmacology-based prediction and analysis. Nucleic Acids Res. 44, W507–W513. 10.1093/nar/gkw335 27131384 PMC4987901

[B77] Hu M. Wang L. Zhang F. Xie Y. Zhang T. Liu H. (2025). Network pharmacology combined with molecular docking and experimental validation of the mechanism of action of columbianetin acetate in the treatment of ovarian cancer. Front. Oncol. 15, 1515976. 10.3389/fonc.2025.1515976 40071097 PMC11894577

[B79] Huang Q. Liu R. Liu J. Huang Q. Liu S. Jiang Y. (2020). Integrated network pharmacology analysis and experimental validation to reveal the mechanism of anti-insulin resistance effects of *Moringa oleifera* seeds. Drug Des. Dev. Ther. 14, 4069–4084. 10.2147/DDDT.S265198 33116398 PMC7539042

[B80] Huang H. Xu J. Zhang S. Zhao J. Liu S. Tian L. (2023). A network pharmacology-based approach to explore the active ingredients and molecular mechanism of shen-kui-tong-mai granules on a rat model with chronic heart failure. J. Pharm. Pharmacol. 75, 764–783. 10.1093/jpp/rgad009 36847133

[B82] Hur H. J. Lee A. S. (2017). Protective effect of Allium tuberosum extract on vascular inflammation in tumor necrosis Factor-α-induced human vascular endothelial cells. J. Cancer Prev. 22, 228–233. 10.15430/JCP.2017.22.4.228 29302580 PMC5751840

[B83] Isshiki K. Asai Y. Tanaka S. Nishio M. Uchida T. Okuda T. (2001). Aurantiamide acetate, a selective cathepsin inhibitor, produced by *Aspergillus penicilloides* . Biosci. Biotechnol. Biochem. 65, 1195–1197. 10.1271/bbb.65.1195 11440138

[B84] Jaafaru M. S. Abd Karim N. A. Enas M. E. Rollin P. Mazzon E. Abdull Razis A. F. (2018). Protective effect of glucosinolates hydrolytic products in neurodegenerative diseases (NDDs). Nutrients 10, 580. 10.3390/nu10050580 29738500 PMC5986460

[B85] Jacques P. F. Cassidy A. Rogers G. Peterson J. J. Meigs J. B. Dwyer J. T. (2013). Higher dietary flavonol intake is associated with lower incidence of type 2 diabetes. J. Nutr. 143, 1474–1480. 10.3945/jn.113.177212 23902957 PMC3743276

[B86] Jean S. Kiger A. A. (2014). Classes of phosphoinositide 3-kinases at a glance. J. Cell Sci. 127, 923–928. 10.1242/jcs.093773 24587488 PMC3937771

[B87] Jennings A. Welch A. A. Spector T. Macgregor A. Cassidy A. (2014). Intakes of anthocyanins and flavones are associated with biomarkers of insulin resistance and inflammation in Women1, 2. J. Nutr. 144, 202–208. 10.3945/jn.113.184358 24336456

[B88] Ji K.-Y. Liu C. Liu Z.-Q. Deng Y.-F. Hou T.-J. Cao D.-S. (2023). Comprehensive assessment of nine target prediction web services: which should we choose for target fishing? Brief. Bioinform 24, bbad014. 10.1093/bib/bbad014 36681902

[B89] Jung U. Choi M.-S. (2014). Obesity and its metabolic complications: the role of adipokines and the relationship between obesity, inflammation, insulin resistance, dyslipidemia and nonalcoholic Fatty liver disease. Int. J. Mol. Sci. 15, 6184–6223. 10.3390/ijms15046184 24733068 PMC4013623

[B90] Kang Y. Kim B.-G. Kim S. Lee Y. Yoon Y. (2017). Inhibitory potential of flavonoids on PtdIns(3,4,5)P3 binding with the phosphoinositide-dependent kinase 1 pleckstrin homology domain. Bioorg. Med. Chem. Lett. 27, 420–426. 10.1016/j.bmcl.2016.12.051 28049590

[B91] Kim S. Chen J. Cheng T. Gindulyte A. He J. He S. (2023). PubChem 2023 update. Nucleic Acids Res. 51, D1373–D1380. 10.1093/nar/gkac956 36305812 PMC9825602

[B93] Knox C. Wilson M. Klinger C. M. Franklin M. Oler E. Wilson A. (2024). DrugBank 6.0: the DrugBank Knowledgebase for 2024. Nucleic Acids Res. 52, D1265–D1275. 10.1093/nar/gkad976 37953279 PMC10767804

[B94] Kolberg L. Raudvere U. Kuzmin I. Adler P. Vilo J. Peterson H. (2023). g:Profiler—interoperable web service for functional enrichment analysis and gene identifier mapping (2023 update). Nucleic Acids Res. 51, W207–W212. 10.1093/nar/gkad347 37144459 PMC10320099

[B95] Kumar L. Vizgaudis W. Klein-Seetharaman J. (2022). Structure‐based survey of ligand binding in the human insulin receptor. Br. J. Pharmacol. 179, 3483–3904. 10.1111/bph.15777 34907529

[B96] Kurup P. A. Rao P. L. N. Venkataraman R. (1954). Antibiotic principle from *Moringa pterygosperma*. VI. Mechanism of antibacterial action of pterygospermin inhibition of transaminase by pterygospermin. Indian J. Med. Res. 42, 115–123. 13162513

[B97] LaMoia T. E. Shulman G. I. (2021). Cellular and molecular mechanisms of metformin action. Endocr. Rev. 42, 77. 10.1210/endrev/bnaa023 32897388 PMC7846086

[B98] Laskowski R. A. Swindells M. B. (2011). LigPlot+: multiple ligand–protein interaction diagrams for drug discovery. J. Chem. Inf. Model. 51, 2778–2786. 10.1021/ci200227u 21919503

[B99] Lebovitz H. E. (2001). Insulin resistance: definition and consequences. Exp. Clin. Endocrinol. Diabetes 109, S135–S148. 10.1055/s-2001-18576 11460565

[B100] Lebovitz H. E. (2019). Thiazolidinediones: the forgotten diabetes medications. Curr. Diabetes Rep. 19, 151. 10.1007/s11892-019-1270-y 31776781 PMC6881429

[B101] Lee M. A. Tan L. Yang H. Im Y.-G. Im Y. J. (2017). Structures of PPARγ complexed with lobeglitazone and pioglitazone reveal key determinants for the recognition of antidiabetic drugs. Sci. Rep. 7, 16837. 10.1038/s41598-017-17082-x 29203903 PMC5715099

[B104] Lin H. Zhu H. Tan J. Wang H. Wang Z. Li P. (2019). Comparative analysis of chemical constituents of *Moringa oleifera* leaves from China and India by ultra-performance liquid chromatography coupled with quadrupole-time-of-flight mass spectrometry. Molecules 24, 942. 10.3390/molecules24050942 30866537 PMC6429208

[B200] Liu H.-X. Lian L. Hou L.-L. Liu C.-X. Ren J.-H. Qiao Y.-B. (2024). Herb pair of Huangqi-Danggui exerts anti-tumor immunity to breast cancer by upregulating *PIK3R1* . Anim. Models Exp. Med. 7, 234–258. 10.1002/ame2.12434 38863309 PMC11228089

[B106] Maliha A. Tahsin M. Fabia T. Z. Rahman S. M. Rahman M. M. (2024). Pro-resolving metabolites: future of the fish oil supplements. J. Funct. Foods 121, 106439. 10.1016/j.jff.2024.106439

[B107] Martin Y. C. (2005). A bioavailability Score. J. Med. Chem. 48, 3164–3170. 10.1021/jm0492002 15857122

[B108] Meng E. C. Goddard T. D. Pettersen E. F. Couch G. S. Pearson Z. J. Morris J. H. (2023). UCSF ChimeraX: tools for structure building and analysis. Protein Sci. 32, e4792. 10.1002/pro.4792 37774136 PMC10588335

[B109] Mohallem R. Aryal U. K. (2020). Regulators of TNFα mediated insulin resistance elucidated by quantitative proteomics. Sci. Rep. 10, 20878. 10.1038/s41598-020-77914-1 33257747 PMC7705713

[B110] Mohanraj K. Karthikeyan B. S. Vivek-Ananth R. P. Chand R. P. B. Aparna S. R. Mangalapandi P. (2018). IMPPAT: a curated database of Indian medicinal plants, phytochemistry and therapeutics. Sci. Rep. 8, 4329. 10.1038/s41598-018-22631-z 29531263 PMC5847565

[B111] Mok T. N. He Q. Zhang X. Sin T. H. Wang H. Hou H. (2022). Effects of 6-hydroxykaempferol: a potential natural product for amelioration of tendon impairment. Front. Pharmacol. 13, 919104. 10.3389/fphar.2022.919104 35935848 PMC9354238

[B112] Molière S. Jaulin A. Tomasetto C.-L. Dali-Youcef N. (2023). Roles of matrix metalloproteinases and their natural inhibitors in metabolism: insights into health and disease. Int. J. Mol. Sci. 24, 10649. 10.3390/ijms241310649 37445827 PMC10341707

[B113] Monteleone M. C. Wusener A. E. G. Burdisso J. E. Conde C. Cáceres A. Arregui C. O. (2012). ER-Bound protein tyrosine phosphatase PTP1B interacts with src at the plasma membrane/substrate interface. PLoS One 7, e38948. 10.1371/journal.pone.0038948 22701734 PMC3372476

[B114] Morris G. M. Huey R. Lindstrom W. Sanner M. F. Belew R. K. Goodsell D. S. (2009). AutoDock4 and AutoDockTools4: automated docking with selective receptor flexibility. J. Comput. Chem. 30, 2785–2791. 10.1002/jcc.21256 19399780 PMC2760638

[B115] Moyo B. Masika P. J. Hugo A. Muchenje V. (2011). Nutritional characterization of moringa (*Moringa oleifera* Lam.) leaves. Afr. J. Biotechnol. 10, 12925–12933. 10.5897/AJB10.1599

[B116] Mthiyane F. T. Dludla P. V. Ziqubu K. Mthembu S. X. H. Muvhulawa N. Hlengwa N. (2022). A review on the antidiabetic properties of *Moringa oleifera* extracts: focusing on oxidative stress and inflammation as main therapeutic targets. Front. Pharmacol. 13, 940572. 10.3389/fphar.2022.940572 35899107 PMC9310029

[B117] Mysinger M. M. Carchia M. Irwin J. J. Shoichet B. K. (2012). Directory of useful decoys, enhanced (DUD-E): better ligands and decoys for better benchmarking. J. Med. Chem. 55, 6582–6594. 10.1021/jm300687e 22716043 PMC3405771

[B118] Nadeem M. Imran M. (2016). Promising features of *Moringa oleifera* oil: recent updates and perspectives. Lipids Health Dis. 15, 1–8. 10.1186/s12944-016-0379-0 27931216 PMC5146848

[B119] Nahm F. S. (2022). Receiver operating characteristic curve: overview and practical use for clinicians. Korean J. Anesthesiol. 75, 25–36. 10.4097/kja.21209 35124947 PMC8831439

[B120] Noor F. Tahir ul Qamar M. Ashfaq U. A. Albutti A. Alwashmi A. S. S. Aljasir M. A. (2022). Network pharmacology approach for medicinal plants: review and assessment. Pharmaceuticals 15, 572. 10.3390/ph15050572 35631398 PMC9143318

[B121] Odermatt A. Arnold P. Stauffer A. Frey B. M. Frey F. J. (1999). The N-terminal anchor sequences of 11β-Hydroxysteroid dehydrogenases determine their orientation in the endoplasmic reticulum membrane. J. Biol. Chem. 274, 28762–28770. 10.1074/jbc.274.40.28762 10497248

[B122] Odermatt A. Atanasov A. G. Balazs Z. Schweizer R. A. S. Nashev L. G. Schuster D. (2006). Why is 11β-hydroxysteroid dehydrogenase type 1 facing the endoplasmic reticulum lumen? physiological relevance of the membrane topology of 11β-HSD1. Mol. Cell. Endocrinol., Int. Workshop 11beta 17beta-Hydroxysteroid Dehydrogenases 248, 15–23. 10.1016/j.mce.2005.11.040 16412558

[B123] Oestlund I. Snoep J. Schiffer L. Wabitsch M. Arlt W. Storbeck K.-H. (2024). The glucocorticoid-activating enzyme 11β-hydroxysteroid dehydrogenase type 1 catalyzes the activation of testosterone. J. Steroid Biochem. Mol. Biol. 236, 106436. 10.1016/j.jsbmb.2023.106436 38035948

[B124] Olivares-Reyes J. A. Arellano-Plancarte A. Castillo-Hernandez J. R. (2009). Angiotensin II and the development of insulin resistance: implications for diabetes. Mol. Cell. Endocrinol. 302, 128–139. 10.1016/j.mce.2008.12.011 19150387

[B125] O’Boyle N. M. Banck M. James C. A. Morley C. Vandermeersch T. Hutchison G. R. (2011). Open babel: an open chemical toolbox. J. Cheminform. 3, 1–14. 10.1186/1758-2946-3-33 21982300 PMC3198950

[B126] O’Connell J. Porter J. Kroeplien B. Norman T. Rapecki S. Davis R. (2019). Small molecules that inhibit TNF signalling by stabilising an asymmetric form of the trimer. Nat. Commun. 10, 5795. 10.1038/s41467-019-13616-1 31857588 PMC6923382

[B127] Park E. J. Kim J. (2007). Cytotoxic sesquiterpene lactones from *Inula britannica* . Planta Med. 64, 752–754. 10.1055/s-2006-957573 9933993

[B128] Park K.-S. Chong Y. Kim M. K. (2016). Myricetin: biological activity related to human health. Appl. Biol. Chem. 59, 259–269. 10.1007/s13765-016-0150-2

[B129] Pathania S. Randhawa V. Bagler G. (2013). Prospecting for novel plant-derived molecules of Rauvolfia serpentina as inhibitors of Aldose reductase, a potent drug target for diabetes and its complications. PLoS One 8, e61327. 10.1371/journal.pone.0061327 23613832 PMC3629236

[B131] Pettersen E. F. Goddard T. D. Huang C. C. Couch G. S. Greenblatt D. M. Meng E. C. (2004). UCSF chimera—A visualization system for exploratory research and analysis. J. Comput. Chem. 25, 1605–1612. 10.1002/jcc.20084 15264254

[B132] Piñero J. Ramírez-Anguita J. M. Saüch-Pitarch J. Ronzano F. Centeno E. Sanz F. (2020). The DisGeNET knowledge platform for disease genomics: 2019 update. Nucleic Acids Res. 48, D845–D855. 10.1093/nar/gkz1021 31680165 PMC7145631

[B134] Qiang G. Xue S. Yang J. J. Du G. Pang X. Li X. (2014). Identification of a small molecular insulin receptor agonist with potent antidiabetes activity. Diabetes 63, 1394–1409. 10.2337/db13-0334 24651808 PMC3964510

[B135] R Core Team (2021). R: a language and environment for statistical computing.

[B136] Raffaele S. Lombardi M. Verderio C. Fumagalli M. (2020). TNF production and release from microglia *via* extracellular vesicles: impact on brain functions. Cells 9, 2145. 10.3390/cells9102145 32977412 PMC7598215

[B137] Rajan T. S. De Nicola G. R. Iori R. Rollin P. Bramanti P. Mazzon E. (2016). Anticancer activity of glucomoringin isothiocyanate in human malignant astrocytoma cells. Fitoterapia 110, 1–7. 10.1016/j.fitote.2016.02.007 26882972

[B138] Rao R. R. George M. Pandalai K. M. (1946). Pterygospermin: the antibacterial principle of *Moringa pterygosperma*, gaertn. Nature 158, 745–746. 10.1038/158745b0 20341204

[B139] Rathinaswamy M. K. Burke J. E. (2020). Class I phosphoinositide 3-kinase (PI3K) regulatory subunits and their roles in signaling and disease. Adv. Biol. Regul. Symp. Issue 75, 100657. 10.1016/j.jbior.2019.100657 31611073

[B140] Reddy K. J. Singh M. Bangit J. R. Batsell R. R. (2010). The role of insulin resistance in the pathogenesis of atherosclerotic cardiovascular disease: an updated review. J. Cardiovasc. Med. 11, 633–647. 10.2459/JCM.0b013e328333645a 20164784

[B141] Ren N. Kim E. Li B. Pan H. Tong T. Yang C. S. (2019). Flavonoids alleviating insulin resistance through inhibition of inflammatory signaling. J. Agric. Food Chem. 67, 5361–5373. 10.1021/acs.jafc.8b05348 30612424

[B142] Robin X. Turck N. Hainard A. Tiberti N. Lisacek F. Sanchez J.-C. (2011). pROC: an open-source package for R and S+ to analyze and compare ROC curves. BMC Bioinform 12, 77. 10.1186/1471-2105-12-77 21414208 PMC3068975

[B143] Romsicki Y. Reece M. Gauthier J.-Y. Asante-Appiah E. Kennedy B. P. (2004). Protein tyrosine phosphatase-1B dephosphorylation of the insulin receptor occurs in a perinuclear endosome compartment in human embryonic kidney 293 cells. J. Biol. Chem. 279, 12868–12875. 10.1074/jbc.M309600200 14722096

[B144] RStudio (2020). RStudio. Boston, MA, United States: Integrated Development for R.

[B145] Saidu Y. Muhammad S. A. Abbas A. Y. Onu A. Tsado I. M. Muhammad L. (2016). *In vitro* screening for protein tyrosine phosphatase 1B and dipeptidyl peptidase IV inhibitors from selected Nigerian medicinal plants. J. Intercult. Ethnopharmacol. 6, 154–157. 10.5455/jice.20161219011346 28512596 PMC5429074

[B146] Saklayen M. G. (2018). The global epidemic of the metabolic syndrome. Curr. Hypertens. Rep. 20, 12. 10.1007/s11906-018-0812-z 29480368 PMC5866840

[B147] Sang S. Lao A. Wang Y. Chin C.-K. Rosen R. T. Ho C.-T. (2002). Antifungal constituents from the seeds of *Allium fistulosum* L. J. Agric. Food Chem. 50, 6318–6321. 10.1021/jf025651o 12381110

[B148] Sashidhara K. V. Rosaiah J. N. Tyagi E. Shukla R. Raghubir R. Rajendran S. M. (2009). Rare dipeptide and urea derivatives from roots of *Moringa oleifera* as potential anti-inflammatory and antinociceptive agents. Eur. J. Med. Chem. 44, 432–436. 10.1016/j.ejmech.2007.12.018 18243423

[B150] Schaffer S. W. Jong C. J. Mozaffari M. (2012). Role of oxidative stress in diabetes-mediated vascular dysfunction: unifying hypothesis of diabetes revisited. Vasc. Pharmacol., Mech. Vasc. Dysfunct. Diabetes 57, 139–149. 10.1016/j.vph.2012.03.005 22480621

[B151] Sfakianos J. Coward L. Kirk M. Barnes S. (1997). Intestinal uptake and biliary excretion of the isoflavone genistein in Rats1,2,3. J. Nutr. 127, 1260–1268. 10.1093/jn/127.7.1260 9202077

[B152] Shamsian S. Sokouti B. Dastmalchi S. (2023). Benchmarking different docking protocols for predicting the binding poses of ligands complexed with cyclooxygenase enzymes and screening chemical libraries. Bioimpacts 14, 29955. 10.34172/bi.2023.29955 38505677 PMC10945300

[B153] Shannon P. Markiel A. Ozier O. Baliga N. S. Wang J. T. Ramage D. (2003). Cytoscape: a software environment for integrated models of biomolecular interaction networks. Genome Res. 13, 2498–2504. 10.1101/gr.1239303 14597658 PMC403769

[B154] Shen H. Pei H. Zhai L. Guan Q. Wang G. (2023). Aurantiamide suppresses the activation of NLRP3 inflammasome to improve the cognitive function and central inflammation in mice with Alzheimer’s disease. CNS Neurosci. Ther. 29, 1075–1085. 10.1111/cns.14082 36627760 PMC10018077

[B155] Shende V. V. Bauman K. D. Moore B. S. (2024). The shikimate pathway: gateway to metabolic diversity. Nat. Prod. Rep. 41, 604–648. 10.1039/d3np00037k 38170905 PMC11043010

[B156] Shu X. Keller T. C. S. Begandt D. Butcher J. T. Biwer L. Keller A. S. (2015). Endothelial nitric oxide synthase in the microcirculation. Cell. Mol. Life Sci. 72, 4561–4575. 10.1007/s00018-015-2021-0 26390975 PMC4628887

[B157] Smith R. A. Baglioni C. (1987). The active form of tumor necrosis factor is a trimer. J. Biol. Chem. 262, 6951–6954. 10.1016/S0021-9258(18)48183-5 3034874

[B158] Sonego P. Kocsor A. Pongor S. (2008). ROC analysis: applications to the classification of biological sequences and 3D structures. Brief. Bioinform. 9, 198–209. 10.1093/bib/bbm064 18192302

[B160] Sorokina M. Merseburger P. Rajan K. Yirik M. A. Steinbeck C. (2021). COCONUT online: collection of open natural products database. J. Cheminform. 13, 2. 10.1186/s13321-020-00478-9 33423696 PMC7798278

[B161] Sourcegraph (2023). Cody AI coding assistant. San Francisco, California, United States: Sourcegraph, Inc.

[B162] Spassov D. S. (2024). Binding affinity determination in drug design: insights from lock and key, induced fit, conformational selection, and inhibitor trapping models. Int. J. Mol. Sci. 25, 7124. 10.3390/ijms25137124 39000229 PMC11240957

[B163] Stein R. M. Yang Y. Balius T. E. O’Meara M. J. Lyu J. Young J. (2021). Property-Unmatched decoys in docking benchmarks. J. Chem. Inf. Model. 61, 699–714. 10.1021/acs.jcim.0c00598 33494610 PMC7913603

[B164] Stelzer G. Rosen N. Plaschkes I. Zimmerman S. Twik M. Fishilevich S. (2016). The GeneCards suite: from gene data mining to disease genome sequence analyses. Curr. Protoc. Bioinform 54 (1.30.1), 1–1.30.33. 10.1002/cpbi.5 27322403

[B165] Stuible M. Tremblay M. L. (2010). In control at the ER: PTP1B and the down-regulation of RTKs by dephosphorylation and endocytosis. Trends Cell Biol. 20, 672–679. 10.1016/j.tcb.2010.08.013 20864346

[B166] Świderska E. Strycharz J. Wróblewski A. Szemraj J. Drzewoski J. Śliwińska A. (2018). “Role of PI3K/AKT pathway in insulin-mediated glucose uptake,” in Blood glucose levels (London, United Kingdom: IntechOpen). 10.5772/intechopen.80402

[B167] Szklarczyk D. Kirsch R. Koutrouli M. Nastou K. Mehryary F. Hachilif R. (2023). The STRING database in 2023: protein–protein association networks and functional enrichment analyses for any sequenced genome of interest. Nucleic Acids Res. 51, D638–D646. 10.1093/nar/gkac1000 36370105 PMC9825434

[B168] Tan P.-N. (2009). “Receiver operating characteristic,” in Encyclopedia of database systems. Editors Liu L. Özsu M. T. (Boston, MA: Springer), 2349–2352. 10.1007/978-0-387-39940-9_569

[B169] Tarkowská D. (2019). Plants are capable of synthesizing animal steroid hormones. Molecules 24, 2585. 10.3390/molecules24142585 31315257 PMC6680614

[B170] The UniProt Consortium (2023). UniProt: the universal protein knowledgebase in 2023. Nucleic Acids Res. 51, D523–D531. 10.1093/nar/gkac1052 36408920 PMC9825514

[B172] Trott O. Olson A. J. (2010). AutoDock Vina: improving the speed and accuracy of docking with a new scoring function, efficient optimization, and multithreading. J. Comput. Chem. 31, 455–461. 10.1002/jcc.21334 19499576 PMC3041641

[B173] Tsay A. Wang J.-C. (2023). The role of PIK3R1 in metabolic function and insulin sensitivity. Int. J. Mol. Sci. 24, 12665. 10.3390/ijms241612665 37628845 PMC10454413

[B175] Vaughn S. F. Berhow M. A. (2005). Glucosinolate hydrolysis products from various plant sources: ph effects, isolation, and purification. Ind. Crops Prod. 21, 193–202. 10.1016/j.indcrop.2004.03.004

[B176] Vergara-Jimenez M. Almatrafi M. M. Fernandez M. L. (2017). Bioactive components in *Moringa oleifera* leaves protect against chronic disease. Antioxidants 6, 91. 10.3390/antiox6040091 29144438 PMC5745501

[B177] Wang F. Zhong H.-H. Chen W.-K. Liu Q.-P. Li C.-Y. Zheng Y.-F. (2016). Potential hypoglycaemic activity phenolic glycosides from *Moringa oleifera* seeds. Nat. Prod. Res. 31, 1869–1874. 10.1080/14786419.2016.1263846 27966373

[B178] Wang J. Zhang M. Sun S. Wan G. Wan D. Feng S. (2021). Network pharmacology-based prediction of catalpol and mechanisms against stroke. Evid.-Based Complement. Altern. Med. 2021, 2541316. 10.1155/2021/2541316 33505489 PMC7810528

[B179] Wang Y. Yang Y. Wang W. Huang Y. Zhang Z. Li J. (2022). Methodology of network pharmacology for research on Chinese herbal medicine against COVID-19: a review. J. Integr. Med. 20, 477–487. 10.1016/j.joim.2022.09.004 36182651 PMC9508683

[B180] Waterman C. Rojas-Silva P. Tumer T. B. Kuhn P. Richard A. J. Wicks S. (2015). Isothiocyanate-rich *Moringa oleifera* extract reduces weight gain, insulin resistance and hepatic gluconeogenesis in mice. Mol. Nutr. Food Res. 59, 1013–1024. 10.1002/mnfr.201400679 25620073 PMC4456298

[B181] Wu J. Zhang F. Li Z. Jin W. Shi Y. (2022). Integration strategy of network pharmacology in Traditional Chinese Medicine: a narrative review. J. Tradit. Chin. Med. 42, 479–486. 10.19852/j.cnki.jtcm.20220408.003 35610020 PMC9924699

[B182] Xiao H.-Y. Li N. Duan J.J.-W. Jiang B. Lu Z. Ngu K. (2020). Biologic-like *in vivo* efficacy with small molecule inhibitors of TNFα identified using scaffold hopping and structure-based drug design approaches. J. Med. Chem. 63, 15050–15071. 10.1021/acs.jmedchem.0c01732 33261314

[B183] Xu H. X. Lee S. F. (2001). Activity of plant flavonoids against antibiotic-resistant bacteria. Phytother. Res. 15, 39–43. 10.1002/1099-1573(200102)15:1<39::aid-ptr684>3.0.co;2-r 11180521

[B184] Yang X. Wong M. Wang N. Sun-Chi Chan A. Yao X. (2006). A new eudesmane derivative and a new fatty acid ester from *Sambucus williamsii* . Chem. Pharm. Bull. 54, 678. 10.1248/cpb.54.676 16651764

[B185] Yang Z.-G. Jia L.-N. Shen Y. Ohmura A. Kitanaka S. (2011). Inhibitory effects of constituents from *Euphorbia lunulata* on differentiation of 3T3-L1 cells and nitric oxide production in RAW264.7 cells. Molecules 16, 8305–8318. 10.3390/molecules16108305 21959301 PMC6264768

[B186] Yang J. Zhou Z. Chen L. Gao L. Lü X. Hong Q. (2023). *In vitro* study on antitumor activity of aurantiamide acetate extracted from *Polygonum capitatum* . S. Afr. J. Bot. 159, 280–289. 10.1016/j.sajb.2023.06.022

[B187] Yildirim M. A. Goh K.-I. Cusick M. E. Barabási A.-L. Vidal M. (2007). Drug-target network. Nat. Biotechnol. 25, 1119–1126. 10.1038/nbt1338 17921997

[B188] Yoon C.-S. Kim D.-C. Lee D.-S. Kim K.-S. Ko W. Sohn J. H. (2014). Anti-neuroinflammatory effect of aurantiamide acetate from the marine fungus *Aspergillus* sp. SF-5921: inhibition of NF-κB and MAPK pathways in lipopolysaccharide-induced mouse BV2 microglial cells. Int. Immunopharmacol. 23, 568–574. 10.1016/j.intimp.2014.10.006 25448500

[B189] Youden W. J. (1950). Index for rating diagnostic tests. Cancer 3, 32–35. 10.1002/1097-0142(1950)3:1<32::aid-cncr2820030106>3.0.co;2-3 15405679

[B190] Yuan J.-W. Hao J. Chen D. (2017). Network pharmacology: an important breakthrough in traditional Chinese medicine research. TMR Integr. Med 2, 92–98. 10.53388/TMRIM201802026

[B191] Zatterale F. Longo M. Naderi J. Raciti G. A. Desiderio A. Miele C. (2020). Chronic adipose tissue inflammation linking obesity to insulin resistance and type 2 diabetes. Front. Physiol. 10, 1607. 10.3389/fphys.2019.01607 32063863 PMC7000657

[B192] Zhai L. Ning Z. Huang T. Wen B. Liao C. Lin C. (2018). Cyclocarya paliurus leaves tea improves dyslipidemia in diabetic mice: a lipidomics-based network pharmacology study. Front. Pharmacol. 9, 973. 10.3389/fphar.2018.00973 30210345 PMC6121037

[B194] Zhang G. Li Q. Chen Q. Su S. (2013). Network pharmacology: a new approach for Chinese herbal medicine research. Evid.-Based Complement. Altern. Med. 2013, e621423. 10.1155/2013/621423 23762149 PMC3671675

[B195] Zhang C. Xu M. He C. Zhuo J. Burns D. M. Qian D.-Q. (2022). Discovery of 1′-(1-phenylcyclopropane-carbonyl)-3H-spiro[isobenzofuran-1,3′-pyrrolidin]-3-one as a novel steroid mimetic scaffold for the potent and tissue-specific inhibition of 11β-HSD1 using a scaffold-hopping approach. Bioorg. Med. Chem. Lett. 69, 128782. 10.1016/j.bmcl.2022.128782 35537608

[B196] Zhao L. Zhang H. Li N. Chen J. Xu H. Wang Y. (2023). Network pharmacology, a promising approach to reveal the pharmacology mechanism of Chinese medicine formula. J. Ethnopharmacol. 309, 116306. 10.1016/j.jep.2023.116306 36858276

[B197] Zhou B. Yang Z. Feng Q. Liang X. Li J. Zanin M. (2017). Aurantiamide acetate from *Baphicacanthus cusia* root exhibits anti-inflammatory and anti-viral effects *via* inhibition of the NF-κB signaling pathway in influenza A virus-infected cells. J. Ethnopharmacol. 199, 60–67. 10.1016/j.jep.2017.01.038 28119097

[B198] Zhu J. Shen L. Shen G. Tao Y. (2024). Optimizing the salt-processing parameters of *Achyranthes bidentata* and their correlation with anti-osteoarthritis effect. Processes 12, 434. 10.3390/pr12030434

[B199] Zou F. Zhao H. Ma A. Song D. Zhang X. Zhao X. (2022). Preparation of an isorhamnetin phospholipid complex for improving solubility and anti-hyperuricemia activity. Pharm. Dev. Technol. 27, 842–852. 10.1080/10837450.2022.2123510 36083162

