# Supplementary material for: Network pharmacology refined with non-ubiquity and decoy-controlled molecular docking reveals insights into Moringa oleifera phytochemicals targeting insulin resistance
Source: Front Bioinform. 2026 Mar 10;6:1756081. doi: 10.3389/fbinf.2026.1756081 (PMC13008888; doi:10.3389/fbinf.2026.1756081)
Supplement: Supplementary file 1 [file DataSheet1.pdf]

Supplementary Material

Supplementary Tables S1-S11 are available as an Excel spreadsheet file:

Supplementary_Tables_S1_to_S11.xlsx

Supplementary Table S12. Examples of target-based assays.

Target	Description	References
HSD11B1	Immuno-radiometric scintillation proximity assay (SPA) of biopsied human adipose tissue treated with ^{3}H-cortisone	(Zhang *et al.*, 2022)
	Immuno-radiometric scintillation proximity assay (SPA) with high-affinity anti-cortisol antibody and ^{3}H-cortisone;	(Sorensen *et al.*, 2006; Li *et al.*, 2018)
INSR	Dissociation-enhanced lanthanide fluorescence immunoassay (DELFIA)	(Treu, 2015)
	Binding Scintillation Proximity Assay (SPA)	(Hundsdörfer *et al.*, 2012)
NOS3	Hemoglobin (Hb) NO capture assay	(Cinelli *et al.*, 2020; Vasu *et al.*, 2025)
	Liquid scintillation spectroscopy with [^{3}H]arginine	(Bredt and Snyder, 1990)
PIK3R1	Homogeneous Time-Resolved Fluorescence (HTRF) assay	(Knight *et al.*, 2010; Erra *et al.*, 2018)
	qPCR-based detection with biotinylated ligand affinity resin	(Zhang, 2019)
PPARA	Transactivation assay for PPAR subtypes	(Henke *et al.*, 1998; Dietz *et al.*, 2012)
PPARG	Binding Scintillation Proximity Assay (SPA)	(Henke *et al.*, 1998)
	Transactivation assay for PPAR subtypes	(Henke *et al.*, 1998; Dietz *et al.*, 2012)
	Lipogenesis assay measuring glucose incorporation into C3H10T1/2 stem cells	(Henke *et al.*, 1998)
PTPN1	Enzyme activity using UV-Vis spectroscopy and p-nitrophenylphosphate substrate	(Huang *et al.*, 2003; Punthasee *et al.*, 2017)

	Enzyme activity using fluorometry	(Scapin *et al.*, 2003; Li *et al.*, 2004)
	Human hepatoma HepG2 cell-based assay	(Boutselis *et al.*, 2007)
	Fragment-based NMR screening	(Black *et al.*, 2005)
TNF	Spectrophotometric enzyme-linked immunosorbent assay (ELISA)	(Pérez *et al.*, 2020)
	Surface Plasmon Resonance (SPR)	(Ma *et al.*, 2014)

Supplementary Table S13. Commercial availability and synthetic accessibility of possible active phytochemicals.

Phytochemical Class	Phytochemical Name	Commercial Availability*	SwissADME Synthetic Accessibility Score (1 = easy to 10 = complex)
Steroid	Boldione	Yes	4.75
Peptide derivative	Aurantiamide acetate	Yes	3.65
	Aurantiamide	No	3.27
Thiocarbamate	Pterygospermin	No	5.16
	O-Ethyl [(3,4-dihydroxyphenyl)methyl] carbamothioate	No	2.03
	O-Methyl N-[(4-hydroxyphenyl)methyl] carbamothioate	No	1.55
Phenolic acid	Ellagic acid	Yes	3.17
Sesquiterpene	4α,6α-Dihydroxyeudesman-8β,12-olide	Yes	4.34
Fatty acid derivative	Sanleng acid	No	4.49
	Tianshic acid	No	4.49
Flavonoid	Luteolin	Yes	3.02
	Scutellarein	Yes	3.04
	Apigenin	Yes	2.96
	Chryseriol	Yes	3.06

	2’,5,5’,7-Tetrahydroxyflavone	Yes	3.09
	Robinetin	Yes	3.21
	Quercetin	Yes	3.23
	6-Hydroxykaempferol	Yes	3.18
	Isorhamnetin	Yes	3.26
	Rhamnetin	Yes	3.30
	2',3,5,7-Tetrahydroxyflavone	Yes	3.24
	Daidzein	Yes	2.79
	Orobol	Yes	2.99
	Genistein	Yes	2.87

* Listing of at least three chemical vendors in PubChem

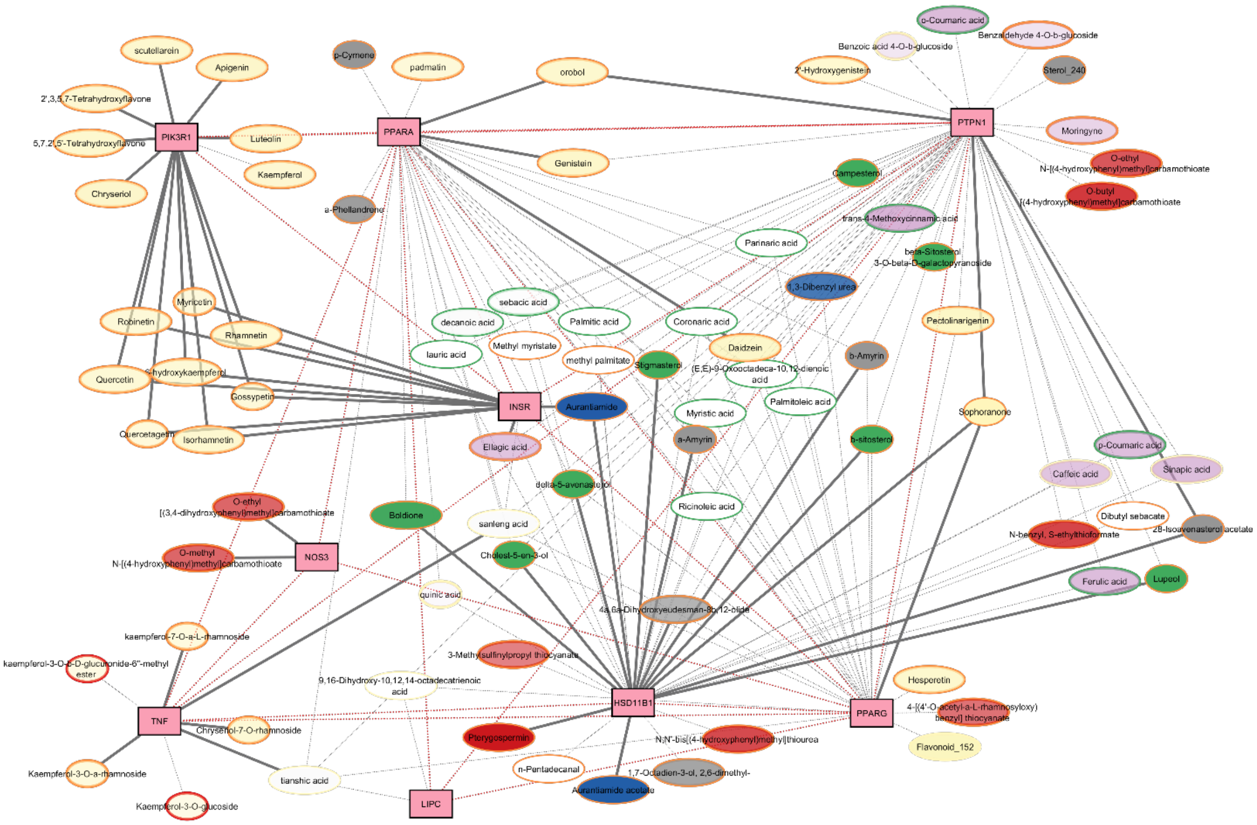

Supplementary Figure S1. Complete phytochemical-protein interaction network including probable nonbinders. Image created using Cytoscape.

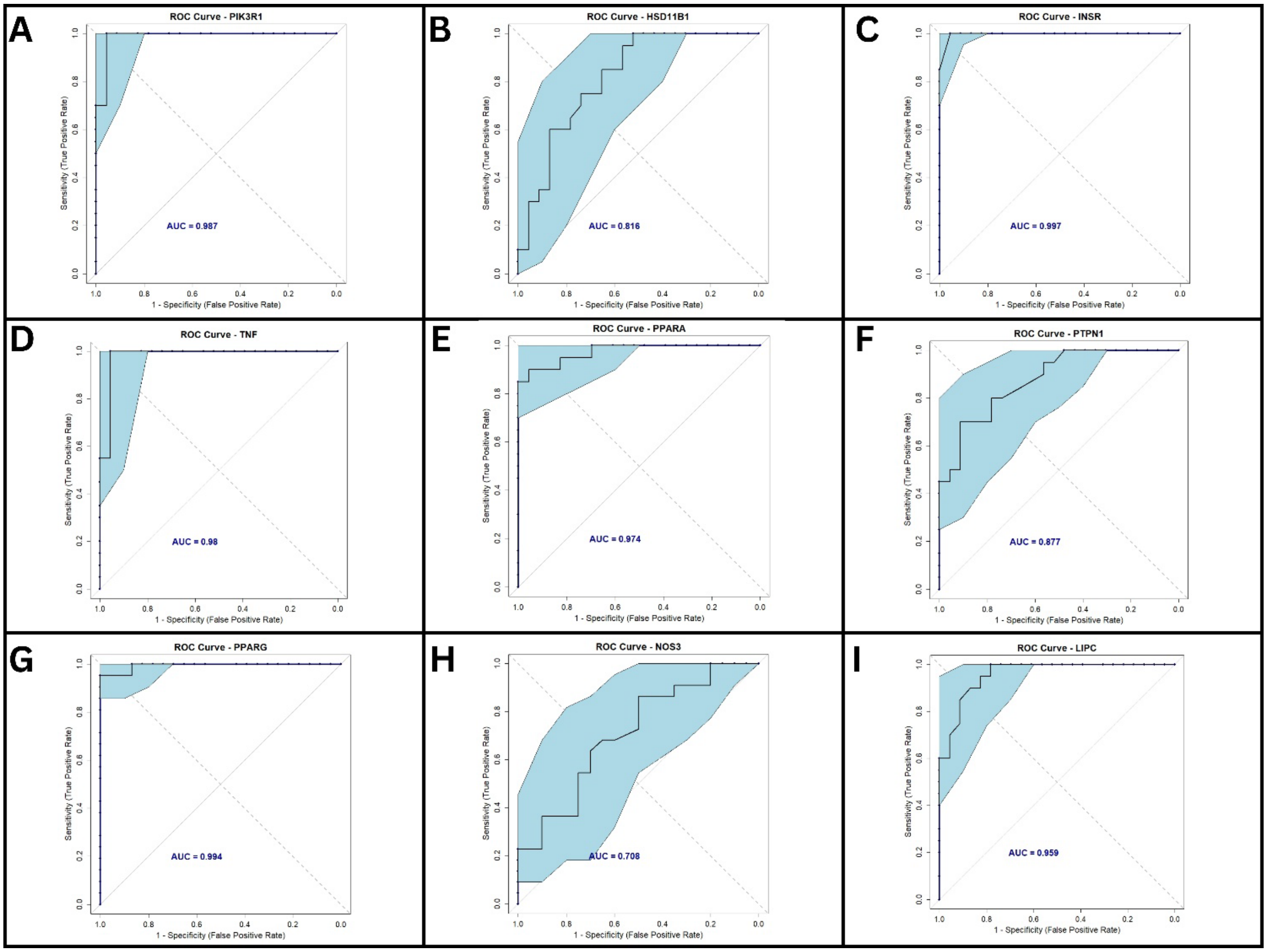

Supplementary Figure S2. Receiver operator characteristic curves to distinguish docking binding affinities of binders versus decoys for each target. All area-under-curve (AUC) values indicate good (AUC ≥ 0.8) or excellent (AUC ≥ 0.9) discrimination between binders and decoys, except for eNOS/NOS3 with fair/moderate discrimination (AUC ≥ 0.7). Images created using pROC in RStudio.

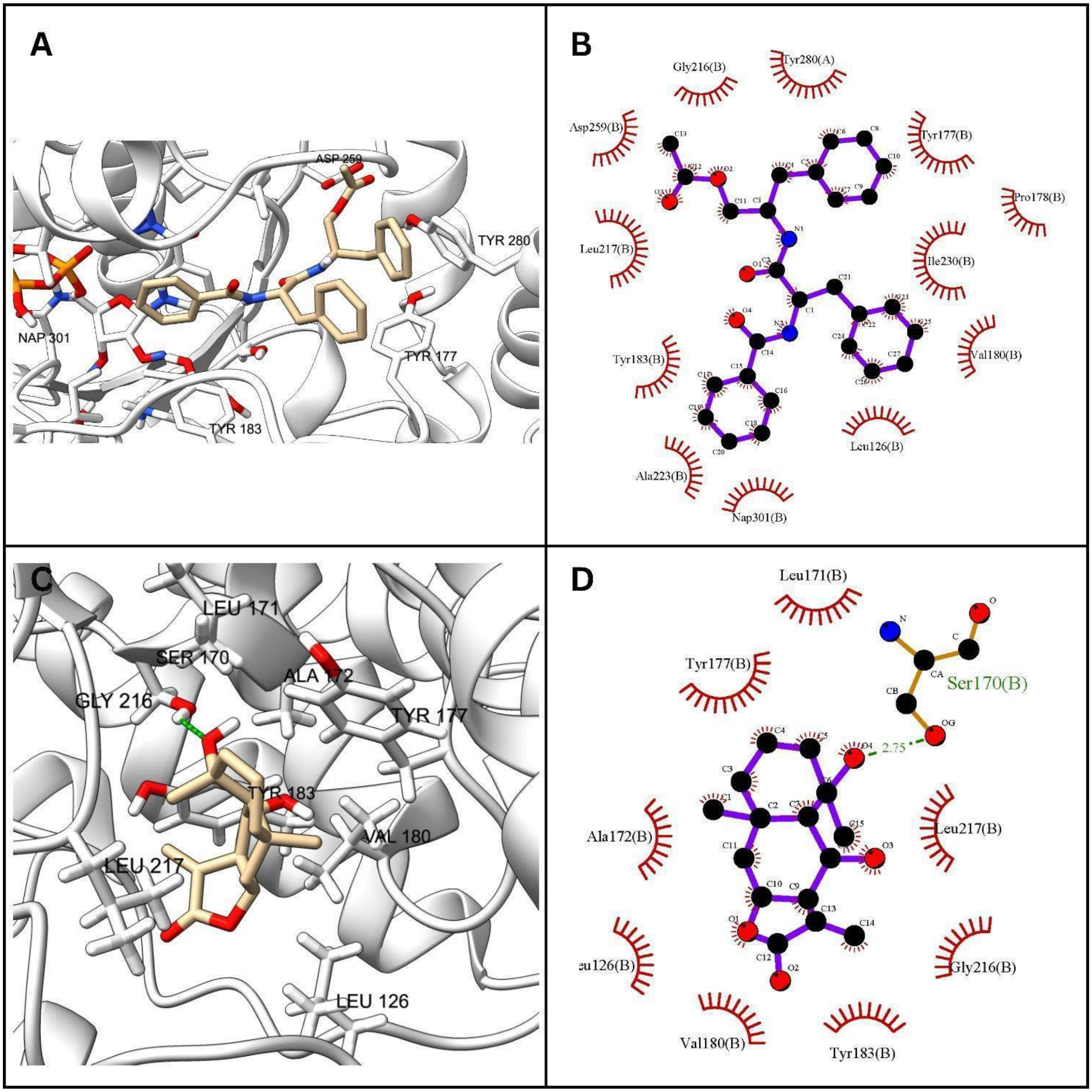

Supplementary Figure S3. Visualization of possible interactions between HSD11B1 and aurantiamide acetate, a peptide derivative (A,B), and 4α,6α-dihydroxyeudesman-8β,12-olide, a sesquiterpenoid (C,D). Interactions with aurantiamide acetate show hydrophobic interactions with catalytic residues Tyr177 and Tyr183 (A,B). The sesquiterpenoid 4α,6α-dihydroxyeudesman-8β,12-olide demonstrates hydrogen bonding with catalytic residue Ser170, as well as hydrophobic interactions with catalytic residues Tyr177 and Tyr183 (C,D). Images created using ChimeraX and LigPlot+.

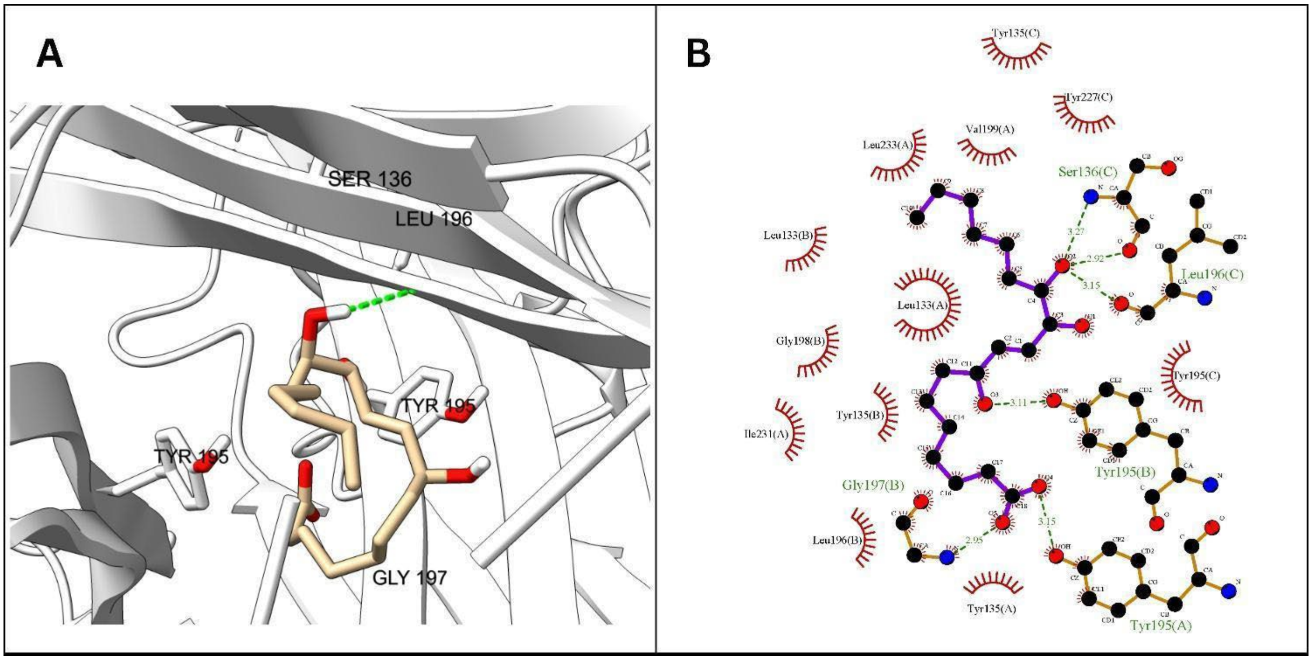

Supplementary Figure S4. Visualization of possible interactions between TNF-α and fatty acid derivative tianshic acid. Allosteric inhibitors of the TNF trimer form hydrogen bonding or hydrophobic interactions with specific tyrosine residues. Images created using ChimeraX and LigPlot+.

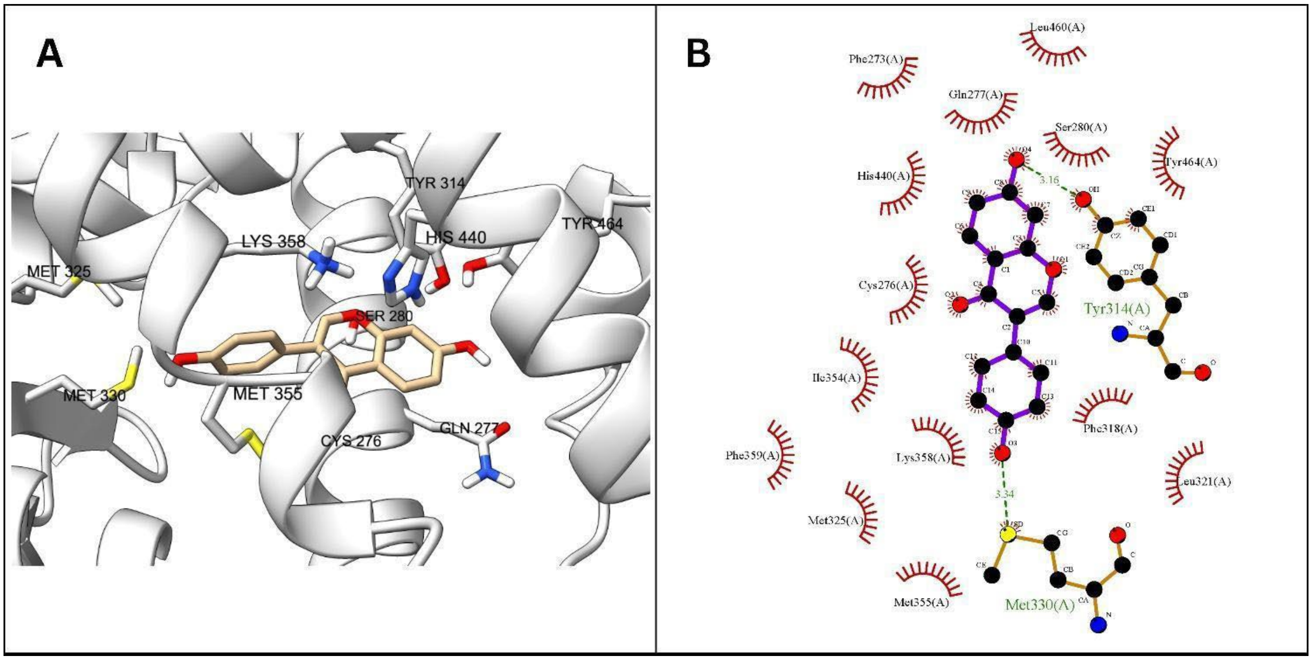

Supplementary Figure S5. Visualization of possible interactions between PPARα and the flavonoid daidzein (A,B). Daidzein exhibits hydrogen bonding with Tyr314, as well as hydrophobic interactions with Tyr464, His440, and Gln277 in the ligand binding domain. Images created using ChimeraX and LigPlot+.

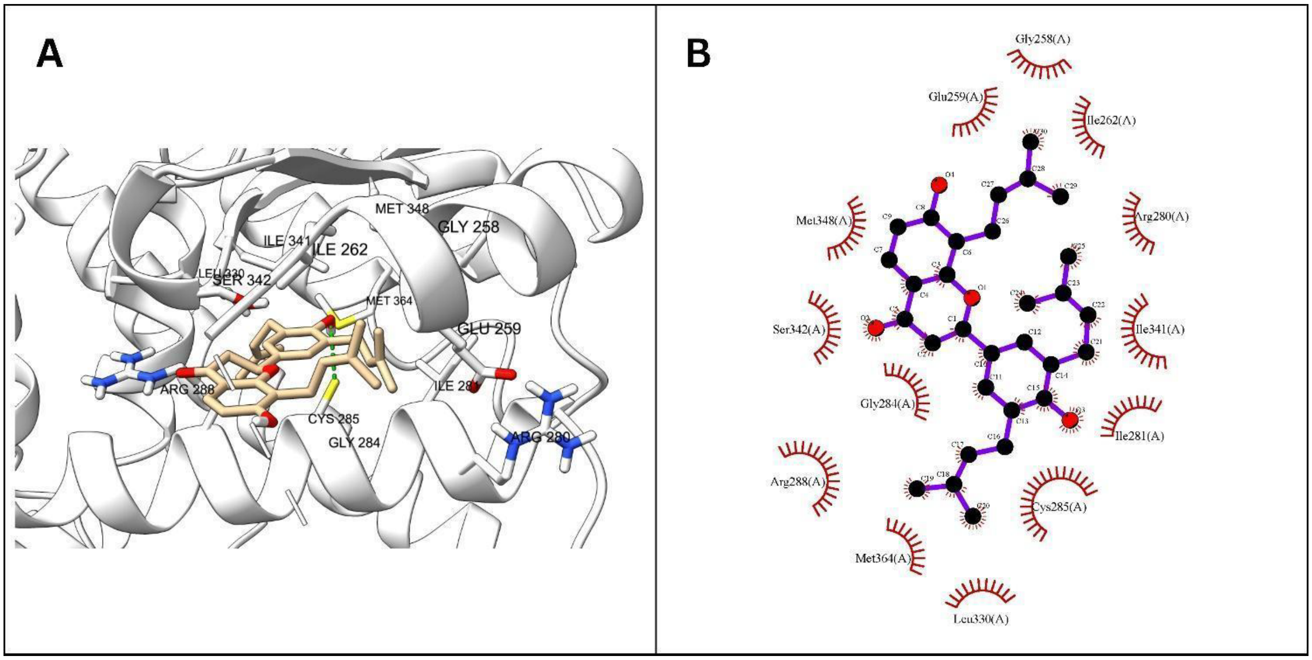

Supplementary Figure S6. Visualization of possible interactions between PPARγ and sophoranone. Sophoranone exhibits hydrophobic interactions with Cys285 in the AF-2 pocket of the ligand-binding domain, the same region where thiazolidinedione drugs form hydrogen bonds. It also forms hydrophobic interactions with Leu330 and Met364 in the central region of the ligand-binding domain. Images created using ChimeraX and LigPlot+.

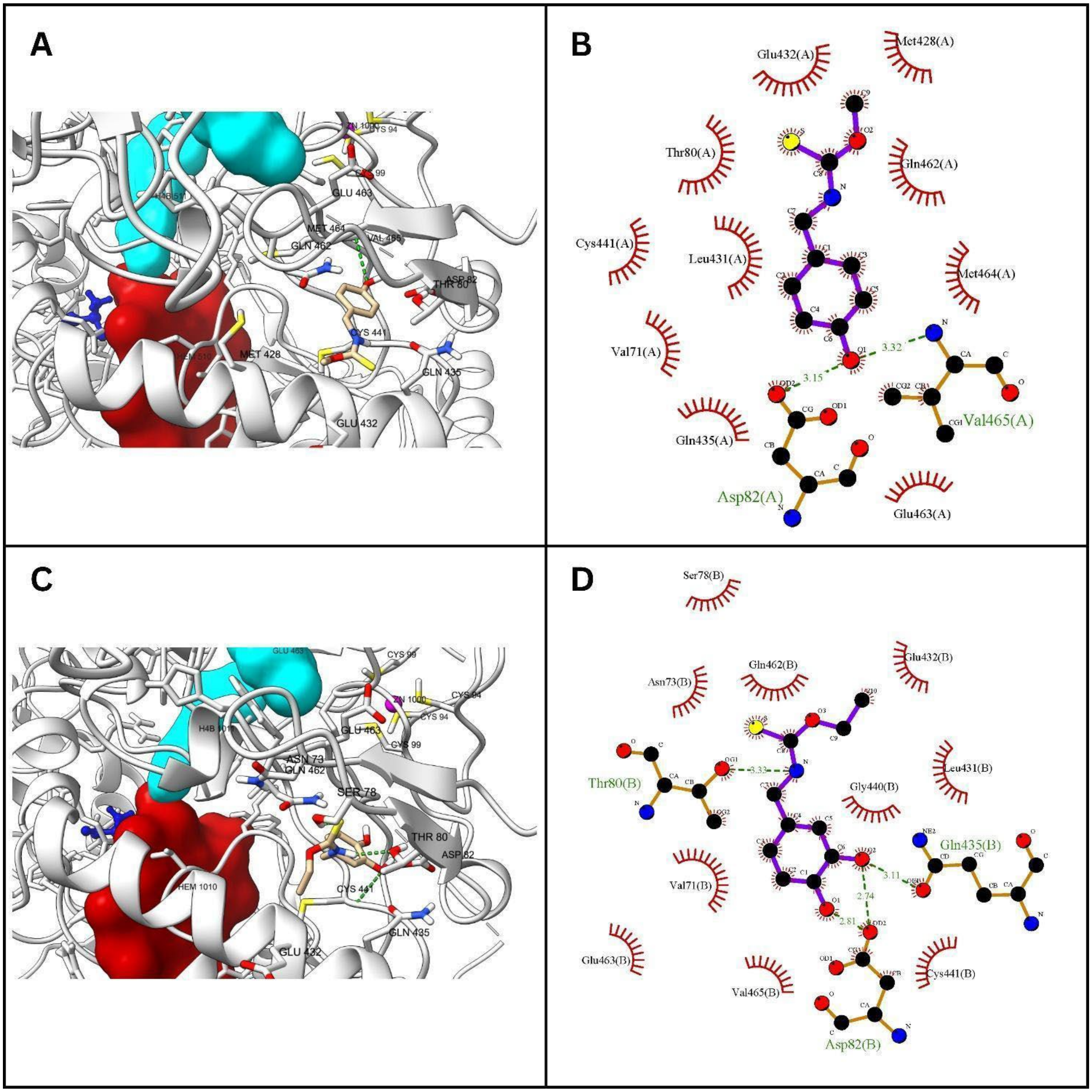

Supplementary Figure S7. Visualization of possible interactions between eNOS and the thiocarbamates O-methyl-N-[(4-hydroxyphenyl)methyl] carbamothioate (**A,B**) and O-ethyl-[(3,4-dihydroxyphenyl)methyl] carbamothioate (**C,D**) showing several interactions with residues in the NOSIP-binding domain comprising residues 366-486. Endothelial NOS exists as a homodimer. The predicted position of the thiocarbamates in the docked structures is between the reductase domain of the dimer pair (not shown) and heme on the oxygenase domain. Cofactors iron-containing heme (HEM, red) and tetrahydrobiopterin (H4B, cyan) are rendered as surface, and the substrate L-arginine is rendered as ball and stick. Coordination of Cys94 and Cys99 residues around zinc is structurally required but does not participate in the electron transfer reaction. Images created using ChimeraX and LigPlot+.

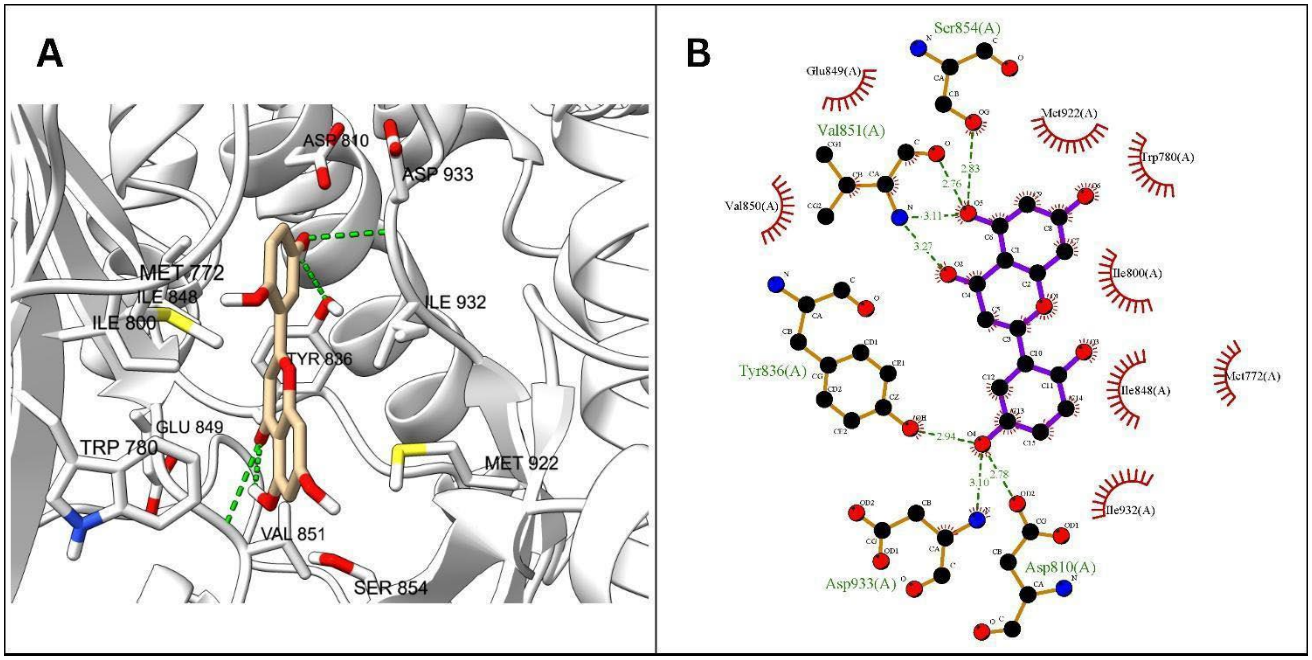

Supplementary Figure S8. Visualization of possible interactions between PIK3R1 and 2’,5,5’,7-tetrahydroxyflavone (A,B) in the ATP binding pocket shows several hydrogen bonding and hydrophobic interactions that were also seen previously in a selective inhibitor of the α isoform. Images created using ChimeraX and LigPlot+.

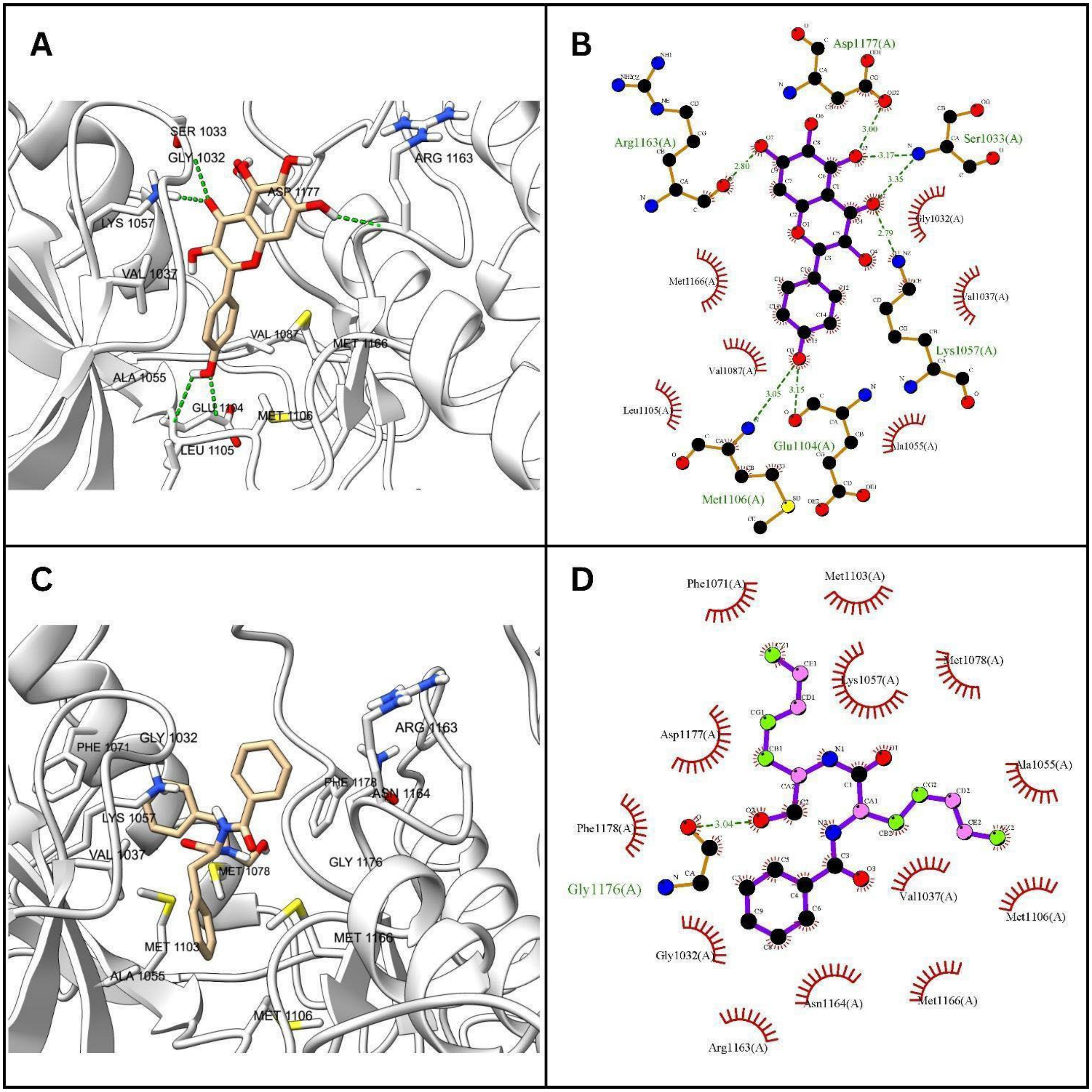

Supplementary Figure S9. Visualization of possible interactions between INSR and 6-hydroxykaempferol (A,B) and aurantiamide (C,D). There are multiple predicted hydrogen bonds between INSR and 6-hydroxykaempferol. For aurantiamide, the hydrogen bond is with Gly1176 adjacent to Asp1177 which is a more common hydrogen bonding site for the cytoplasmic inhibitor binding pocket. Images created using ChimeraX and LigPlot+.

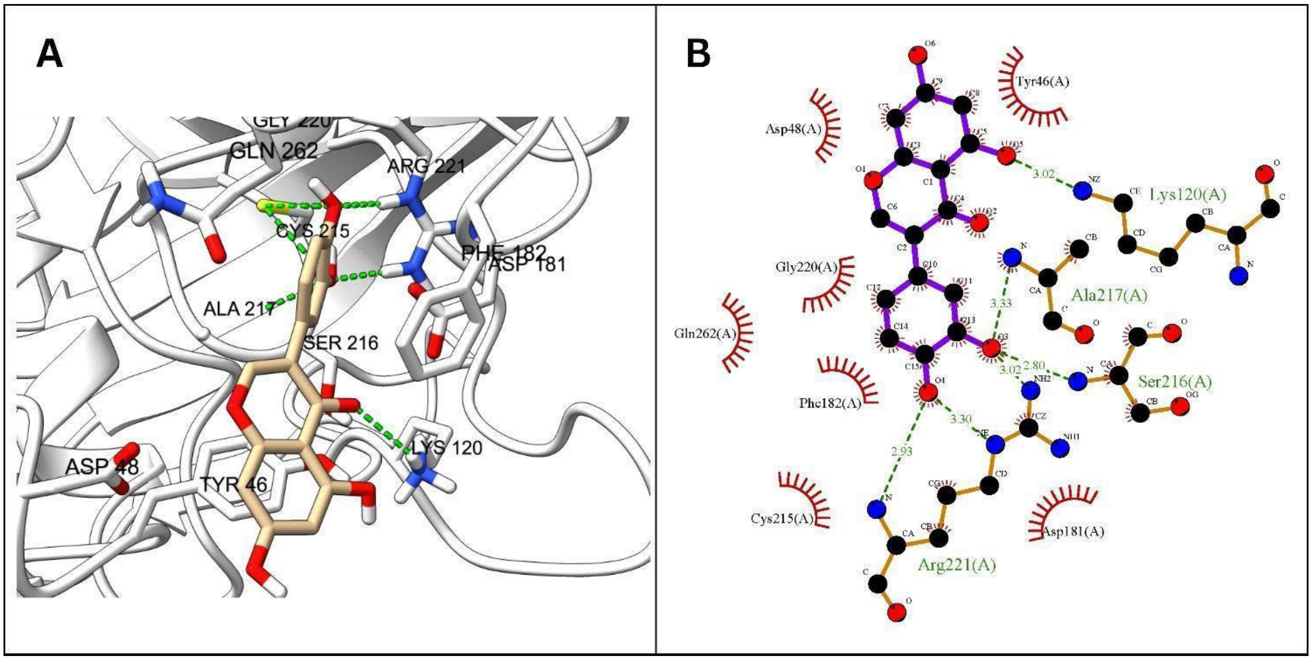

Supplementary Figure S10. Visualization of possible interactions between PTPN1 and orobol shows hydrogen bonding with the catalytic residue Cys215, substrate binding site Arg221, and proton donor Asp181. Images created using ChimeraX and LigPlot+.

References

Black, E. *et al.* (2005) "Structure-based design of protein tyrosine phosphatase-1B inhibitors," *Bioorganic & Medicinal Chemistry Letters*, 15(10), pp. 2503–2507. Available at: https://doi.org/10.1016/j.bmcl.2005.03.068.

Boutselis, I.G. *et al.* (2007) "Synthesis and Cell-Based Activity of a Potent and Selective Protein Tyrosine Phosphatase 1B Inhibitor Prodrug," *Journal of Medicinal Chemistry*, 50(4), pp. 856–864. Available at: https://doi.org/10.1021/jm061146x.

Bredt, D.S. and Snyder, S.H. (1990) "Isolation of nitric oxide synthetase, a calmodulin-requiring enzyme.," *Proceedings of the National Academy of Sciences*, 87(2), pp. 682–685. Available at: https://doi.org/10.1073/pnas.87.2.682.

Cinelli, M.A. *et al.* (2020) "First Contact: 7-Phenyl-2-Aminoquinolines, Potent and Selective Neuronal Nitric Oxide Synthase Inhibitors That Target an Isoform-Specific Aspartate," *Journal of Medicinal Chemistry*, 63(9), pp. 4528–4554. Available at: https://doi.org/10.1021/acs.jmedchem.9b01573.

Dietz, M. *et al.* (2012) "Comparative Molecular Profiling of the PPARα/γ Activator Aleglitazar: PPAR Selectivity, Activity and Interaction with Cofactors," *ChemMedChem*, 7(6), pp. 1101–1111. Available at: https://doi.org/10.1002/cmdc.201100598.

Erra, M. *et al.* (2018) "Discovery of a Novel Inhaled PI3Kδ Inhibitor for the Treatment of Respiratory Diseases," *Journal of Medicinal Chemistry*, 61(21), pp. 9551–9567. Available at: https://doi.org/10.1021/acs.jmedchem.8b00873.

Henke, B.R. *et al.* (1998) "N-(2-Benzoylphenyl)-l-tyrosine PPARγ Agonists. 1. Discovery of a Novel Series of Potent Antihyperglycemic and Antihyperlipidemic Agents," *Journal of Medicinal Chemistry*, 41(25), pp. 5020–5036. Available at: https://doi.org/10.1021/jm9804127.

Huang, P. *et al.* (2003) "Structure-Based design and discovery of novel inhibitors of protein tyrosine phosphatases," *Bioorganic & Medicinal Chemistry*, 11(8), pp. 1835–1849. Available at: https://doi.org/10.1016/S0968-0896(03)00039-7.

Hundsdörfer, C. *et al.* (2012) "Indeno[1,2-*b*]indole derivatives as a novel class of potent human protein kinase CK2 inhibitors," *Bioorganic & Medicinal Chemistry*, 20(7), pp. 2282–2289. Available at: https://doi.org/10.1016/j.bmc.2012.02.017.

Knight, S.D. *et al.* (2010) "Discovery of GSK2126458, a Highly Potent Inhibitor of PI3K and the Mammalian Target of Rapamycin," *ACS Medicinal Chemistry Letters*, 1(1), pp. 39–43. Available at: https://doi.org/10.1021/ml900028r.

Li, J. *et al.* (2018) "Discovery of Clinical Candidate BMS-823778 as an Inhibitor of Human 11β-Hydroxysteroid Dehydrogenase Type 1 (11β-HSD-1)," *ACS Medicinal Chemistry Letters*, 9(12), pp. 1170–1174. Available at: https://doi.org/10.1021/acsmedchemlett.8b00307.

Li, X. *et al.* (2004) "α,α-Difluoro-β-ketophosphonates as potent inhibitors of protein tyrosine phosphatase 1B," *Bioorganic & Medicinal Chemistry Letters*, 14(16), pp. 4301–4306. Available at: https://doi.org/10.1016/j.bmcl.2004.05.082.

Ma, L. *et al.* (2014) “A Novel Small-molecule Tumor Necrosis Factor α Inhibitor Attenuates Inflammation in a Hepatitis Mouse Model *,” *Journal of Biological Chemistry*, 289(18), pp. 12457–12466. Available at: https://doi.org/10.1074/jbc.M113.521708.

Pérez, V.T. *et al.* (2020) “Synthesis and biological screening of a library of macamides as TNF-α inhibitors,” *RSC Medicinal Chemistry*, 11(10), pp. 1196–1209. Available at: https://doi.org/10.1039/D0MD00208A.

Punthasee, P. *et al.* (2017) “Covalent Allosteric Inactivation of Protein Tyrosine Phosphatase 1B (PTP1B) by an Inhibitor–Electrophile Conjugate,” *Biochemistry*, 56(14), pp. 2051–2060. Available at: https://doi.org/10.1021/acs.biochem.7b00151.

Scapin, G. *et al.* (2003) “The Structural Basis for the Selectivity of Benzotriazole Inhibitors of PTP1B,” *Biochemistry*, 42(39), pp. 11451–11459. Available at: https://doi.org/10.1021/bi035098j.

Sorensen, B. *et al.* (2006) “Adamantane 11-β-HSD-1 inhibitors: Application of an isocyanide multicomponent reaction,” *Bioorganic & Medicinal Chemistry Letters*, 16(23), pp. 5958–5962. Available at: https://doi.org/10.1016/j.bmcl.2006.08.129.

Treu, M. (2015) “5,8-dihydro-6H-pyrazolo[3,4-h]quinazolines as IGF-1R/IR inhibitors.” Ingelheim am Rhein. Available at: https://patents.google.com/patent/US9150578B2/en (Accessed: January 8, 2026).

Vasu, D. *et al.* (2025) “Truncated pyridinylbenzylamines: Potent, selective, and highly membrane permeable inhibitors of human neuronal nitric oxide synthase,” *Bioorganic & Medicinal Chemistry*, 124, p. 118193. Available at: https://doi.org/10.1016/j.bmc.2025.118193.

Zhang, C. *et al.* (2022) “Discovery of 1′-(1-phenylcyclopropane-carbonyl)-3H-spiro[isobenzofuran-1,3′-pyrrolidin]-3-one as a novel steroid mimetic scaffold for the potent and tissue-specific inhibition of 11β-HSD1 using a scaffold-hopping approach,” *Bioorganic & Medicinal Chemistry Letters*, 69, p. 128782. Available at: https://doi.org/10.1016/j.bmcl.2022.128782.

Zhang, D. (2019) “Pyridine compounds used as PI3 kinase inhibitors.” Suzhou. Available at: https://patents.google.com/patent/US10173995B2/en (Accessed: January 8, 2026).
